# Modeling the effect of temperature and relative humidity on exposure to SARS‐CoV‐2 in a mechanically ventilated room

**DOI:** 10.1111/ina.13146

**Published:** 2022-11-27

**Authors:** Timothy G. Foat, Benjamin Higgins, Charlotte Abbs, Thomas Maishman, Simon Coldrick, Adrian Kelsey, Matthew J. Ivings, Simon T. Parker, Catherine J. Noakes

**Affiliations:** ^1^ Defence Science and Technology Laboratory Salisbury UK; ^2^ Health and Safety Executive Derbyshire Buxton UK; ^3^ Leeds Institute for Fluid Dynamics, School of Civil Engineering University of Leeds Leeds UK

**Keywords:** aerosol, computational fluid dynamics, cough, COVID‐19, exhalation, exposure

## Abstract

Computational fluid dynamics models have been developed to predict airborne exposure to the SARS‐CoV‐2 virus from a coughing person in a mechanically ventilated room. The models were run with three typical indoor air temperatures and relative humidities (RH). Quantile regression was used to indicate whether these have a statistically significant effect on the airborne exposure. Results suggest that evaporation is an important effect. Evaporation leads to respiratory particles, particularly those with initial diameters between 20 and 100 μm, remaining airborne for longer, traveling extended distances and carrying more viruses than expected from their final diameter. In a mechanically ventilated room, with all of the associated complex air movement and turbulence, increasing the RH may result in reduced airborne exposure. However, this effect may be so small that other factors, such as a small change in proximity to the infected person, could rapidly counter the effect. The effect of temperature on the exposure was more complex, with both positive and negative correlations. Therefore, within the range of conditions studied here, there is no clear guidance on how the temperature should be controlled to reduce exposure. The results highlight the importance of ventilation, face coverings and maintaining social distancing for reducing exposure.

**Practical implications**

- The following are based on the study of a coughing person in a generic mechanically ventilated room and the fluid dynamics of COVID‐19 transmission only.
- Higher exposures are likely within 2 m of a person, therefore, face coverings, social distancing and ventilation are important.
- Setting the humidity in a mechanically ventilated room to a high, but comfortable, level (50%–70% RH) may slightly reduce the inhaled dose.
- Ventilation should not be compromised to achieve higher RH and consideration should be given to the increase in the probability of fomite transmission.
- For the range of conditions studied here, there is no clear evidence that the temperature should be controlled in a normal indoor environment to reduce exposure, therefore, it can be set to a comfortable level.

## 1 INTRODUCTION

For COVID‐19 and other respiratory diseases, indoor spaces may present a high‐hazard environment when infection can be transmitted by exhaled, virus carrying, aerosol and droplets. Smaller aerosol particles present an ongoing hazard, as they can remain airborne for long periods of time. Larger aerosols and droplets can evaporate to smaller sizes and subsequently remain airborne, be inhaled directly, deposit onto mucous membranes, or onto exposed surfaces. Early in the COVID‐19 pandemic, advice was being given to maintain social distancing and manage fomite (contaminated surface) risks through good hand hygiene. As knowledge of transmission developed, advice to ventilate spaces while avoiding air recirculation and to wear face coverings was an increasing focus. Advice on ventilation aimed to address the aerosol hazard, social distancing primarily the large droplet hazards, and face coverings the transmission risk from both. However, the behavior of aerosols and droplets released during exhalations is complex and more recent evidence shows that small aerosols are also important for close‐range exposure.^1^ Understanding this complexity, including the role of evaporation of respiratory aerosols and droplets under different indoor environmental conditions is important for explaining transmission mechanisms and providing effective public health advice.

The evaporation rate of aerosols and droplets is primarily dependent on the water content and chemical composition of the droplet (and subsequently the vapor pressure), the local temperature and relative humidity (RH) and other factors, such as the molecular diffusion coefficient of the vapor. A number of studies, discussed below, have looked at droplet evaporation and transport and the general conclusion is that lower temperature and/or higher relative humidity leads to a slower rate of evaporation and therefore larger droplets at a given time. These larger droplets will stay suspended in the air for less time and therefore present less of an airborne hazard.

The classic Wells evaporating‐falling droplet curve^2^ shows that falling droplets smaller than a critical diameter (around 100 –140 μm) will evaporate completely before they reach the ground. Wells hypothesized that this created “droplet nuclei”, which were aerosols <5 μm diameter that could carry microorganisms in the air for long periods of time. Droplets larger than the critical diameter will reach the ground before they can evaporate significantly and do so increasingly rapidly with increasing initial diameter. Wells' work was revisited by Xie et al.,^3^ who built a more complete model of exhaled droplet evaporation and motion. Similarly to Wells, Xie et al. showed that the critical diameter increased as the relative humidity decreased. Xie et al. showed how the picture is even more complex, particularly when considering an exhaled jet containing droplets. They found that there were three critical diameters. For droplets ≤40 μm diameter, an increase in RH resulted in the droplet moving further horizontally before evaporating; for droplets with diameters around 60 μm, this effect was reversed (i.e., an increase in RH resulted in droplets traveling less far before evaporation); and for droplets ≥80 μm there was almost no effect from a change in the RH. Neither Wells' nor Xie et al.’s droplets contained solids, so could evaporate completely, unlike real respiratory droplets.

Wang et al.^4^ also considered evaporating droplets in an exhaled jet but did not consider the temperature difference between the ambient air and exhaled air or the radial velocity component. They did, however, reach the broadly supported conclusion (Xie et al.^3^) that high RH results in slower evaporation and larger droplets deposit more quickly, which in turn reduces the airborne hazard. A more recent study of the evaporation of single droplets by Chen^5^ showed that while for a stationary droplet, an increase in RH always results in an increase in droplet lifetime (note that this is for a stationary droplet, not a falling droplet), an increase in temperature does not always have the opposite effect. They reported that increased temperature only decreased the lifetime when the RH was below a critical threshold (37% in their case, where they consider 20 and 37°C).

A number of studies have used computational fluid dynamics (CFD) to examine the effect of temperature and RH on exhaled droplets. Li et al.^6^ used a Reynolds averaged Navier–Stokes (RANS) approach to model water droplets with non‐volatile cores in an unventilated room; their simulation ran for 30 s. They concluded that increased droplet evaporation could lead to the increased probability of infection. For the conditions, they considered (RH from 10% to 90% and temperature from 3 to 35°C; these upper and lower limits are outside comfortable indoor ranges), a change in RH was more important than a change in temperature when it came to evaporation (evaporation time or time to hit the floor).

Li et al.^7^ also used a RANS approach in an outdoor environment and droplets where the vapor pressure was reduced as a result of the presence of salts. They concluded that for smaller droplets (24 μm initial diameter), changing the RH from 60% to 90% did not have a significant effect on lifetime or distance traveled. Their data, which was only shown for times up to 8 s, did show an effect from RH, but the change was not monotonic. For larger droplets (100–1000 μm) the effect was larger, with the biggest effect (lower RH results in further distances) occurring for diameters between approximately 125 and 300 μm. These droplet diameter boundaries are different from those of Xie et al.^3^

That Xie et al. did not show an RH effect for the largest droplets is perhaps due to the different resolution and scope of the two models (i.e., simple model vs CFD and quiescent environment vs windy/turbulent environment). The switching from RH increasing distances traveled for smaller sizes, to the opposite effect for intermediate sizes, that Xie et al. showed, is perhaps hinted at in Li et al.’s data^7^ for 24 μm droplets. Xie et al.’s droplets were pure water so could evaporate fully, so the results may not be expected to match the smaller droplets. This comparison highlights the advantages of both modeling methods. An analytical model allows for the transition between phases to be more easily differentiated, while a CFD model allows more realistic features to be included for example, complex ventilation airflow.

Yang et al.^8^ modeled dispersion of exhaled droplets having non‐volatile cores within an air‐conditioned bus, using RANS CFD. They showed that for 50 μm diameter droplets, increasing the RH from 35% to 95% (a very high RH for an air‐conditioned space) resulted in the evaporation time (to the solid core) increasing from 1.8 to 7.0 s. However, the effect of RH on dispersion distance was not significant.

High‐resolution CFD modeling of exhaled droplets has also been published. Chong et al.^9^ carried out direct numerical simulation (DNS) of exhaled pure water droplets in a quiescent environment with a temperature of 20°C and RH between 50% and 90% (also a very high RH for an air‐conditioned space). They reported an increase in droplet lifetimes (i.e., time to full evaporation) with an increase in RH. Interestingly, they also reported that the exhaled puff could be sustained for longer distances when the ambient RH was higher.

There is also some empirical evidence to support the effect of RH on both airborne and surface deposited concentration. Parhizkar et al.^10^ carried out experiments with subjects, who had been diagnosed with COVID‐19, carrying out a range of activities in an exposure chamber. They reported that lower RH (over the range of 20%–70%) resulted in higher airborne and lower surface viral concentrations (as measured using polymerase chain reaction). However, their airborne concentration data does appear to be quite skewed by many high C_T_ (cycle threshold) data (i.e., low viral concentration values).

In the current work reported here, a RANS CFD‐based stochastic droplet transport model has been produced for a coughing person in a typical mechanically ventilated meeting room or office space. Coughing was chosen over other types of exhalation as it is a particularly common symptom of COVID‐19. However, it is recognized that speaking for an extended period of time might produce a larger volume of exhaled droplets than a single or even multiple coughs. This work aims to show whether the temperature or RH effects reported for simpler models (analytical or more simplified CFD models) are still present when realistic room airflows are included and exposures are calculated over 5 min time‐scales. The study is primarily focused on the fluid dynamics effects of a change in temperature and RH. It has already been demonstrated that temperature and RH can affect both the survival of airborne and surface‐deposited viruses^11^ and affect the physiology of both the infected and susceptible people, but these factors are not being considered here. By studying the fluid dynamics effects in isolation, the aim is to be able to show whether these need to be taken into account when choosing optimum parameters from a viral decay or physiological perspective.

The work builds on our CFD modeling in Coldrick et al.,^12^ where we validated the CFD methodology for exhaled droplet transport and deposition using experimental data for bacteria collected from speaking, singing and coughing human subjects in an exposure chamber. A statistical assessment of CFD model results has been used to see whether temperature and/or RH have a significant effect on the likely viral exposure. The statistical presentation of the results, that is, the possible range of the received exposures and the statistical significance of any correlations, make this work different from most previous work. Reporting the results in terms of viral exposure rather than droplet number or mass (as has been done in much of the previous work), enables overall risk to be estimated.

## 2 METHODS

### 2.1 The room and scenarios

**FIGURE 1:**
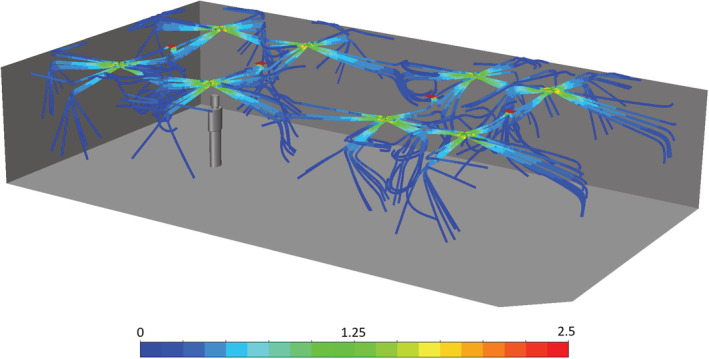
The coughing person standing in the meeting room. The extract vents are shown in red. Pathlines from the eight supply vents are shown, colored by velocity (m·s^−1^).

The effect of temperature and RH on exposure to the SARS‐CoV‐2 virus was studied in a representative mechanically ventilated meeting room/office space, based on a room studied in Foat et al.^13^ The room, chosen as a generic example, was 13.0 long, 7.0 wide and 2.6 m high with a small cut‐out in one corner. The room volume was approximately 237 m^3^ and had mixing ventilation. The air was supplied through eight diffusers and extracted through four, which were all located on the ceiling. All ceiling diffusers were square, four‐way diffusers (the effective air discharge area of each diffuser was 0.0446 m^2^). It was assumed that the air change rate was 5 h^−1^ and that there was no recirculation. The room contained no furniture. A single coughing person was standing in the room, 3 m from one wall, and was facing along the long axis of the room, towards the centre of the room (see Figure 1).

Nine models were run with all combinations of three temperatures and three relative humidities. The temperatures, 16, 20 and 28°C, were chosen to represent: the minimal permissible temperature,^14^ a typical temperature for an air‐conditioned room and an upper limit^15^ respectively. The relative humidities: 30%, 50% and 70%, were chosen to represent likely ranges in mechanically ventilated offices. 30% is often given as a lower comfortable limit and 30%, 50% and 70% were used by Xie et al.^3^

### 2.2 The model

The CFD model used an unsteady RANS approach, with a two‐way coupled Lagrangian phase for the exhaled droplets. This approach has been applied many times previously for similar studies.^6^, ^7^, ^8^, ^16^, ^17^ Other studies have used large‐eddy simulation^18^ (LES) and even DNS^9^. While LES and DNS may be able to better resolve the details of the exhaled plume, it was not practical to use these high‐resolution approaches for the scenarios considered here, that is, whole room scale for 5 min.

The shear‐stress transport (SST) turbulence model^19^ was used, as it has been widely applied to indoor airflows and was used by Coldrick et al.^12^ A coupled solver was used for the pressure–velocity coupling, second‐order schemes were used for all the convection terms and a second‐order implicit scheme was used for the temporal discretization. Buoyancy effects was modeled by solving the energy equation along with the incompressible ideal gas assumption. Only convective heat transfer was included. The simulations were carried out using ANSYS® Fluent® version 2019 R2.

#### 2.2.1 Modeling the droplets

The exhaled droplets were modeled as water droplets with a non‐volatile fraction, so their evaporation rate was based on the vapor pressure of pure water, and their size was reduced until only the solid non‐volatile core remains. This is a simplification from real exhaled droplets, which consist of a complex mixture of salts, proteins and surfactants.^20^ It has already been demonstrated that water droplets can evaporate more rapidly than saliva or saline droplets^21^ and can follow different trajectories as a result. The vapor pressure of saliva droplets is lower than that of pure water and will decrease as the concentration of the salts in the droplet increases.^21^ The non‐volatile mass fraction was set to 1.25%, based on the exhaled particle composition given by Stettler et al.,^21^ which is also close to that in Walker et al.^21^ (2.1%). The results will be strongly dependent on the non‐volatile mass fraction, with a lower mass fraction enabling droplets to evaporate further, so allowing larger initial diameter droplets, containing more virus, to stay airborne.

This simplification was made to reduce computing overhead with the rationale that all but the largest droplets evaporate to their equilibrium state quickly, whether they are water or saliva. Walker et al.^21^ showed that the largest saliva droplets they considered, 200 μm diameter, reached their equilibrium in 60 s when exhaled into 20°C and 50% RH air and 100 μm droplets took less than 20 s. As the larger droplets sediment to the floor quickly (Xie et al.^3^ showed that droplet with diameters ≥100 μm hit the ground in less than approximately 16 s), it was decided that this was a reasonable simplification to make.

The droplet transport and the mass and heat exchange between the droplets and the bulk phase was modeled as described by Coldrick et al.,^12^ with the exception that the work presented here treated the droplets as being composed of water with a non‐volatile core, whereas Coldrick et al. treated them as a multi‐component mixture. The particle force balance included the drag force and gravity only, with the drag force being determined from the mean and turbulent flow. The droplet transport used a discrete random walk (DRW) model. It is known that the DRW walk model, particularly when combined with an isotropic turbulence model (such as SST), can give poor predictions for deposition rates for certain scenarios and particles sizes^23^ (specifically smaller particles). However, as the deposition of the virus will be dominated by sedimentation of the larger droplets, this was not expected to be an issue.

The secondary break‐up was not considered due to the low Weber number of the droplets^24^ (maximum of 1.5). The effects of Brownian motion were also not included. This is because it has been suggested^25^ that the effect is only significant for particles with diameters ≤0.03 μm, which is smaller than the particles considered in the current study. The smallest droplet modeled in this work had an initial diameter of 0.25 μm and a final diameter, after evaporation, of 0.06 μm. The decay in the viability of the virus in droplets was not considered as part of this work.

#### 2.2.2 The computational geometry and mesh

The room and air supply and extract vents are described in Sections 2.1 and 2.2.3. The coughing person was represented by a simplified geometry, which was based on anthropometric data for a female.^26^ They were 1.63 m tall, with their mouth centred on 1.43 m high. The mouth was represented by a circle with a diameter of 2.25 cm, based on the mouth opening area given by Gupta et al.^27^

**FIGURE 2:**
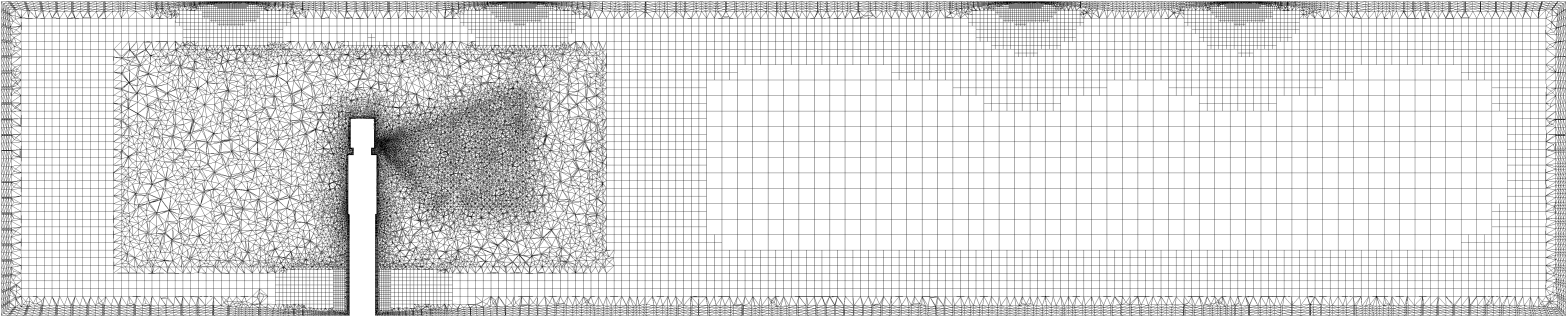
Mesh on a vertical plane through the centre of the mouth.

The geometry was meshed using unstructured tetrahedral cells in a region containing the person's head and upper body, with hex‐core in the rest of the room, see Figure 2. The mesh was refined around the mouth and the exhaled jet and around the supply and extract vents. A mesh sensitivity study was conducted and the results of this are given in Section 2.6. The total cell count was 3.2 million, and the average y^+^ (the non‐dimensional near‐wall cell distance) on the body surface was between 4.1 and 4.4 depending on the temperature and RH.

#### 2.2.3 Boundary conditions

The supply vents were defined as mass‐flow inlets with the air entering the room at 30° to the horizontal, with 5% turbulent intensity and a length scale of 0.01 m. The temperature and RH of the incoming were set to match the conditions for the specific simulation. The extract vents were set as pressure outlets.

It was assumed that the person was fully clothed, so only their convective heat flux was modeled. This was applied as a surface heat flux of 25 W m^−2^. This value is similar to that measured by Zhu et al.^28^ for a resting subject and was used in Coldrick et al.^12^ The heat flux was assumed to be the same for all the temperature and RH conditions. All walls, the floor and the ceiling were given adiabatic boundary conditions. A constant body heat flux for all ambient temperature conditions resulted in the body surface temperature increasing (by approximately 11°C) as the ambient temperature increased. The speed of the thermal plume up the body was similar for all ambient temperatures, with an average peak velocity of approximately 0.3 m·s^−1^ (measured at the top of the torso). Due to the strength of the mechanical ventilation in the room, the thermal plume did not have much effect on the room airflow, apart from close to the person.

The mouth was a velocity inlet with a time‐varying velocity profile and droplet source term as defined in the following section. The temperature and RH of the exhaled air were also specified at the mouth, see Table 1.

**TABLE 1:** Flow properties for the cough exhalation

Parameter	Value	Ref
Mouth diameter/m	0.0225	27
Jet angle θ_1_/degrees	15	27
Jet angle θ_2_/degrees	40	27
Jet angle φ/degrees	90	27
Duration/s	0.4	27
Peak time/s	0.08	27
Peak velocity/m·s^−1^	15	27
Temperature/°C	34	21
RH	100	21

### 2.3 The exhalation

Each simulation consisted of five coughs, with each cough followed by 5 min of mixing. The particles from each cough were deleted at the end of the mixing period. This was done to enable the average effect of the five coughs to be calculated. A simulation was run to see whether the 5 min mixing period was sufficient to capture the bulk of the exposure for a person standing close to the infected person. This work is described in more detail in the Supplementary information S1. In summary, the exposures within 3 m in front of the infected person rise rapidly in the first minute and then continue to increase more slowly out to 5 min and beyond. Therefore, the results presented here do not show the full exposure that a person might receive if they were to stay in the room for 30 min.

**FIGURE 3:**
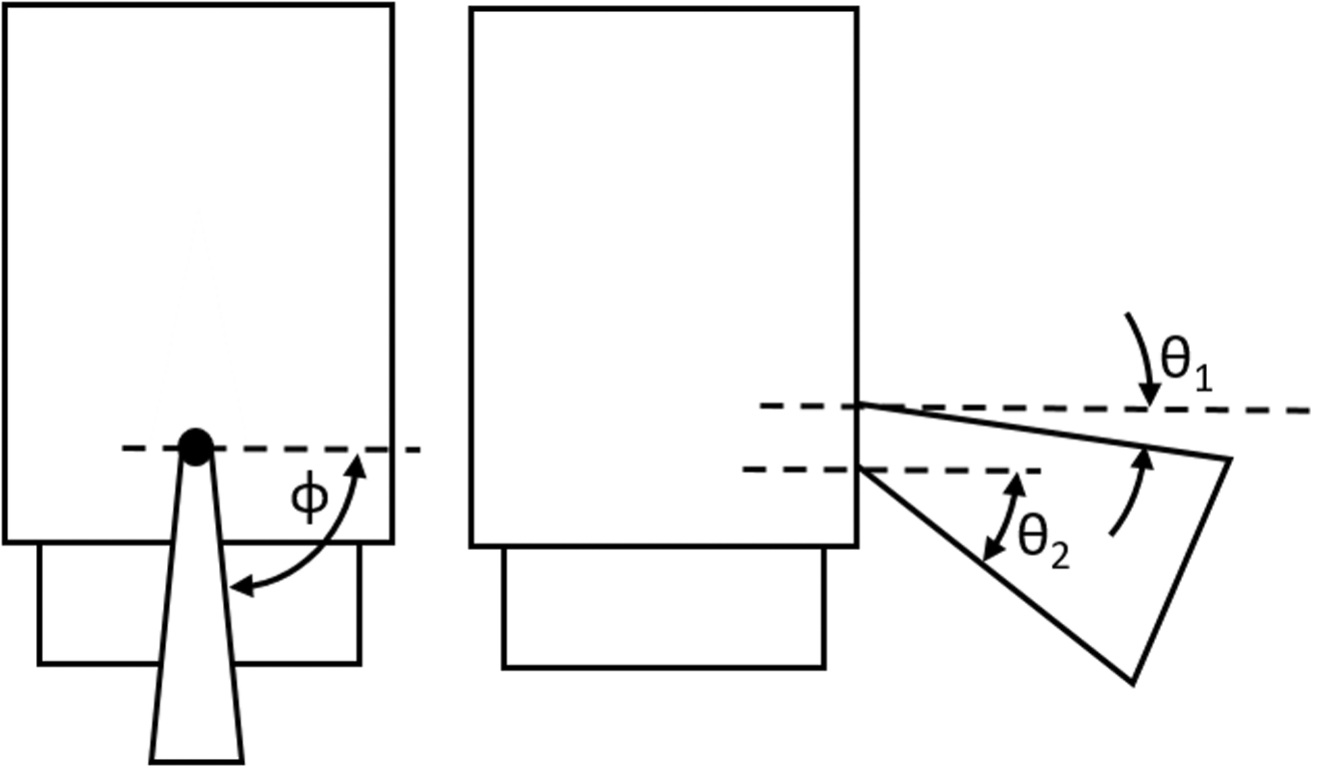
Initial jet expansion angles for the cough, viewed from the front and side.^27^

Only the exhalation part of the cough was modeled, which was approximated as a triangular velocity profile having a duration of 0.4 s and a peak velocity of 15 m·s^−1^ at 0.08 s, based on Gupta et al.^27^ The carrier flow velocity was specified over the mouth as defined by Gupta et al.^27^ and as shown in Figure 3. The turbulence intensity and length scale were set to 10% and 0.01 m respectively. The flow properties for the cough are shown in Table 1. During the mixing period after each cough, there was no air movement from the mouth (i.e., no breathing).

As in Coldrick et al.,^12^ the bronchiolar, laryngeal and oral (BLO) model^29^ was used to describe the distribution of exhaled droplets. The BLO model describes the droplet size distribution for using a tri‐modal distribution fitted to experimental measurements of particles from coughing. The parameters for BLO droplet size distribution are given in Table 2.

**TABLE 2:** Parameters for the BLO model for coughing.^29^

	Mode 1, bronchiolar	Mode 2, laryngeal	Mode 3, oral
Geometric mean diameter/μm	1.57	1.60	123.3
Geometric standard deviation	1.25	1.68	1.84
Total number concentration/cm^−3^	0.0903	0.142	0.0160

The exhaled droplets were distributed across random locations over the mouth and at a random time point during the exhalation as described in Coldrick et al.^12^ The number of droplets emitted during each time step was determined by the fraction of the total volume exhaled during that time interval. Over the duration of the exhalation, the sampled distribution approached the specified BLO distribution.

In Lagrangian particle tracking, each computational particle can represent a parcel of droplets. This is usually done to reduce the total number of particles that need to be simulated. However, for the BLO coughing model, only 310 droplets were exhaled per cough. As this was not likely to provide a statistically significant number of droplets, ten times as many droplets were tracked per cough (3093 in total), with each droplet carrying one‐tenth the viral RNA copies. This approach might have an effect on the extreme ends of the exposure distributions. Each simulation then included five coughs, to further increase the number of droplets and to account for the uncertainty caused by the varying airflow in the room. The sensitivity of the model to the number of coughs, and subsequently the number of droplets is discussed in Section 2.6 and the Supplementary Information S1.

The BLO‐fitted size distribution consisted of 21 logarithmically spaced bins from 0.25 to 398 μm diameter. However, due to the shape of the BLO distribution, two of the intermediate‐size bins (10 and 14.5 μm diameter) contained no droplets. A list of the size bins is given in the Supplementary Information S1.

Different descriptions for exhaled droplet size distributions exist and many of these have been reviewed by Pöhlker et al.^30^ It may be that the trough between the B and L modes and the O mode in the Johnson et al.^29^ model has been exaggerated by the aerosol measurement instruments used.

It is reasonable to ignore the hazard from the droplets exhaled from breathing during the mixing period because the total volume of exhaled droplets over 5 min of breathing is approximately 600 times less than that from a single cough (based on breathing data from Stettler et al^22^).

### 2.4 Simulation strategy

The flow in the room was first solved as steady‐state, then the model was run using an unsteady solver for 290 s with Δ*t* = 1 and 10 s with Δ*t* = 0.1. To capture the dynamics of the cough and the initial droplet transport, the model was then run for 10 s with Δ*t* = 0.01 s. The time step size was then reduced to 1 s from the remainder of the simulation. The sensitivity of the results to a change in Δ*t* was assessed, and the findings are discussed in Section 2.6.

### 2.5 Analysis methods

#### 2.5.1 Calculating exposure

The viral exposure, *E* (viral RNA copies·s·m^‐3^), was calculated in the model to show how this parameter changes as a function of temperature and RH and distance from the person. The mean expected number, *μ*, of viral RNA copies in a droplet was estimated using initial droplet diameter, *d*_0_ [m], and viral load, *c*_*v*_ [RNA copies·m^−3^], using Equation (1).^31^ The number of droplets represented by each tracked parcel (one‐tenth in this case) was also taken into account.

(1) $$\mu =\frac{\pi }{6}d_{0}^{3}c_{v}$$

The SARS‐CoV‐2 viral load value used in this study was 2.76 × 10^9^ copies·ml^−3^ (2.76 × 10^15^ copies·m^−3^). This figure represents an average of peak viral loads over time.^32^ Details of how this figure was produced are given in the Supplementary Information S1. For this viral load, a droplet with an initial diameter of 8.8 μm will have an expected mean number of viral RNA copies equal to one and it will be increasingly likely that smaller droplets will contain no RNA as *d*_0_ reduces.

This study has focused on the exposure to SARS‐CoV‐2 virus, but the relative effects predicted may be applied in principle to any respiratory virus with a viral load that is constant across the range of droplet sizes.

Exposures were calculated within sub‐volumes (as a post‐processing step) using Equation (2).

(2) $$E=\sum_{i}^{N}\frac{\mu_{i}t_{i}}{V}$$

where *N* is the number of droplets passing through the sub‐volume, *t* (s) is the time each particle spends in the sub‐volume and *V* (m^3^) is the volume of the sub‐volume.

The size of the sub‐volume for the main analysis was (0.125 m)^3^, that is, approximately 2 L. This was based on an assumed region from which a person could draw breath. Other studies have used similar^33^ and larger volumes.^34^ For the contour plots, a larger volume was used: (0.2 m)^3^.

Particles were free to recirculate through volumes and sub‐volumes, so can contribute to the exposure multiple times. This approach does not account for a person inhaling and retaining the particles, so may over‐estimate the exposure. It is only the small particles that are likely to recirculate so this effect should be minimal for the overall exposure, which is dominated by larger particles.

Published CFD studies use both the exposure‐based approach applied here^33^, ^34^ and an explicit representation of a breathing susceptible person.^35^ Both methods have their advantages and disadvantages and these are discussed in the Supplementary Information S1.

### 2.6 Validation and model sensitivities

A number of tests were conducted to ensure that the simulations were conducted in a way that reduced the likelihood of producing an inaccurate or misleading solution. All tests used the distributions of exposure (e.g., see Figure 10) as the variable of interest.

For the mesh sensitivity study, the mesh was coarsened by increasing all cell sizes by a factor of 1.25 (resulting in 2.3 million cells) and was refined by reducing all sizes by a factor of 0.5 (resulting in 12.6 million cells). The mesh sensitivity results are shown in the Supplementary Information S1. Analysis showed that there was some dependence of the exposure distributions to the mesh. This was complicated by variability between results on the same mesh, which was a function of the limited number of coughs and droplets that could be modeled for practical reasons. The variability/dependence appeared to be greatest furthest from the person, where the mesh was coarser. This variability reduces the level of confidence in the absolute magnitude of the CFD exposure predictions when using the standard mesh, which was the one that was taken forward in this study.

For the time step dependency, all time step sizes were either multiplied or divided by a factor of two to produce the coarse and refined resolution simulations. The time step sensitivity results are shown in the Supplementary Information S1. As with the mesh dependency, there was some dependence of the exposure distributions on the time step size. However, the change in the median exposure was relatively small when changing from the standard to the short‐time step sizes, particularly further from the person. For this reason, the standard time step sizes were used in the current study.

A model was also run with ten coughs to see whether more coughs (and subsequently more droplets) affected the exposure predictions. The results are shown in the Supplementary Information S1. Analysis showed that the exposure distributions had not converged fully after five coughs and that it took between six and eight coughs for a more converged solution, depending on the distance from the person. As the exposure distributions solution are not fully converged, the results of this work should be considered a snapshot of what could happen for any five coughs, rather than a perfect statistically stationary solution.

#### 2.6.1 Validation

The model was validated using a range of data. To give confidence in the overall predictions from the model, sub‐components were compared with experimental data for the velocity decay in a turbulent jet, the evaporation of a falling droplet and airflow and temperatures around a thermal mannequin. For the current study, the methodology, geometry and mesh were based, where possible, on that used by Coldrick et al.,^12^ who validated their model against experimental data for deposited and airborne bacteria from speaking, singing and coughing human subjects.

In addition to the validation outlined above, predictions for the dispersion of a tracer gas in the meeting room being studied here (see Section 2.1) were compared to data from an experiment described in Foat et al.^13^ The model was run with the same settings as described at the start of Section 2.2, but with steady‐state airflow and isothermal conditions, as there were no heat sources in the room during the experiment. The CFD model did not capture some of the unsteadiness in the concentration field, as would be expected when using a steady RANS modeling approach, but it captured the trends well. More details are given in the Supplementary Information S1.

The purpose of this modeling study was to predict the general effect of temperature and RH on the dispersion of exhaled droplets in a mechanically ventilated meeting room. The full‐model and sub‐component validation work, together with the model sensitivity assessments have demonstrated that the modeling method applied here is fit‐for‐purpose.

## 3 RESULTS AND DISCUSSION

### 3.1 Flow fields

The airflow into the room is illustrated in Figure 1. This shows how the air enters the room from the eight supply vents. The air flows along the ceiling before being deflected down when it impacts on either another supply jet or a wall. The coughing person was placed in the centre of the room width‐wise and this was located approximately below the convergence of two supply jets. This may result in more unsteadiness in the flow in this region compared to other parts of the room.

### 3.2 Droplet/particle transport

**FIGURE 4:**
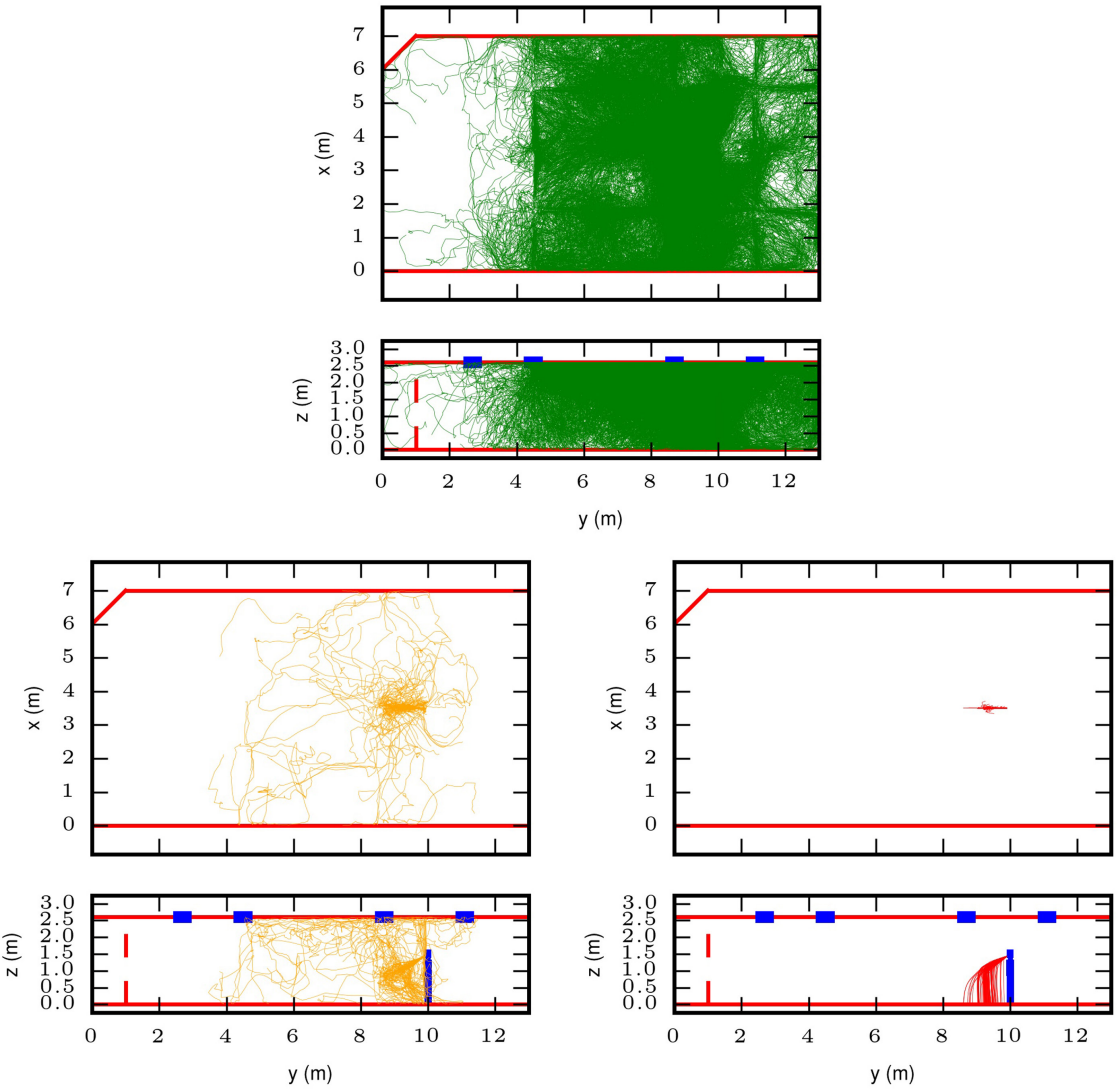
Droplet/particle tracks for three size bins: d_0_ ≤ 20 μm (upper), 20 μm < d_0_ ≤ 100 μm (lower left), d_0_ > 100 μm (lower right). Tracks are shown for one cough only, for the highest evaporation, hot and dry case (T = 28°C and RH = 30%). A plan view and side‐elevation is shown for each size bin. The red solid or dashed lines indicate the walls of the room and the blue rectangles are the extract vents.

Droplet/particle tracks are shown in Figure 4. The tracks are shown for three size bins: d_0_ ≤ 20 μm, 20 μm < d_0_ ≤ 100 μm, d_0_ > 100 μm. These bins were chosen to represent the small aerosol, the intermediate sizes and the large ballistic droplets respectively. The tracks are for one cough only for the highest evaporation, hot and dry case (T = 28°C and RH = 30%). It should be noted that there were far fewer particles in the two larger diameter size bins compared to the smallest bin.

The tracks show that the smallest size droplets/particles mix across large parts of the room resulting in a fairly uniform distribution across two‐thirds of the room. For the data shown here, the ventilation in the room has minimized the smallest droplets mixing, to any large extent, into the far side of the room. The largest sizes follow a ballistic path, as expected. Different sizes travel different distances depending on their initial diameter, however all deposit within 2 m. The intermediate sizes are the most complex; these are carried in the jet of exhaled air and often evaporate sufficiently to remain airborne over distances of more than 2 m.

The plan view for the smallest droplets/particles in Figure 4 shows how they have been advected towards the ceiling and then follow the airflow from the four‐way air supply diffusers. The intermediate sizes can be seen here to be drawn into the thermal plume around the person and carried up to the top of the room. This effect is apparent in the droplet/particle tracks for two of the high‐temperature cases (RH = 30% and 50%), but it is not clear whether this is due to the droplets evaporating more rapidly in these cases or simply due to the random nature of the flow and particle tracks in the room.

Due to the unsteadiness in the room airflow, the droplet/particle tracks from two coughs for the same temperature and RH conditions can look very different. Some exhaled droplets mix almost symmetrically across the room, while droplets/particles from another cough travel more to one side of the room. It may be that the location where the coughing person was placed is particularly susceptible to unsteady airflow. It would be interesting, in future studies, to place the infected person in different locations to see whether the results are sensitive to this change.

**FIGURE 5:**
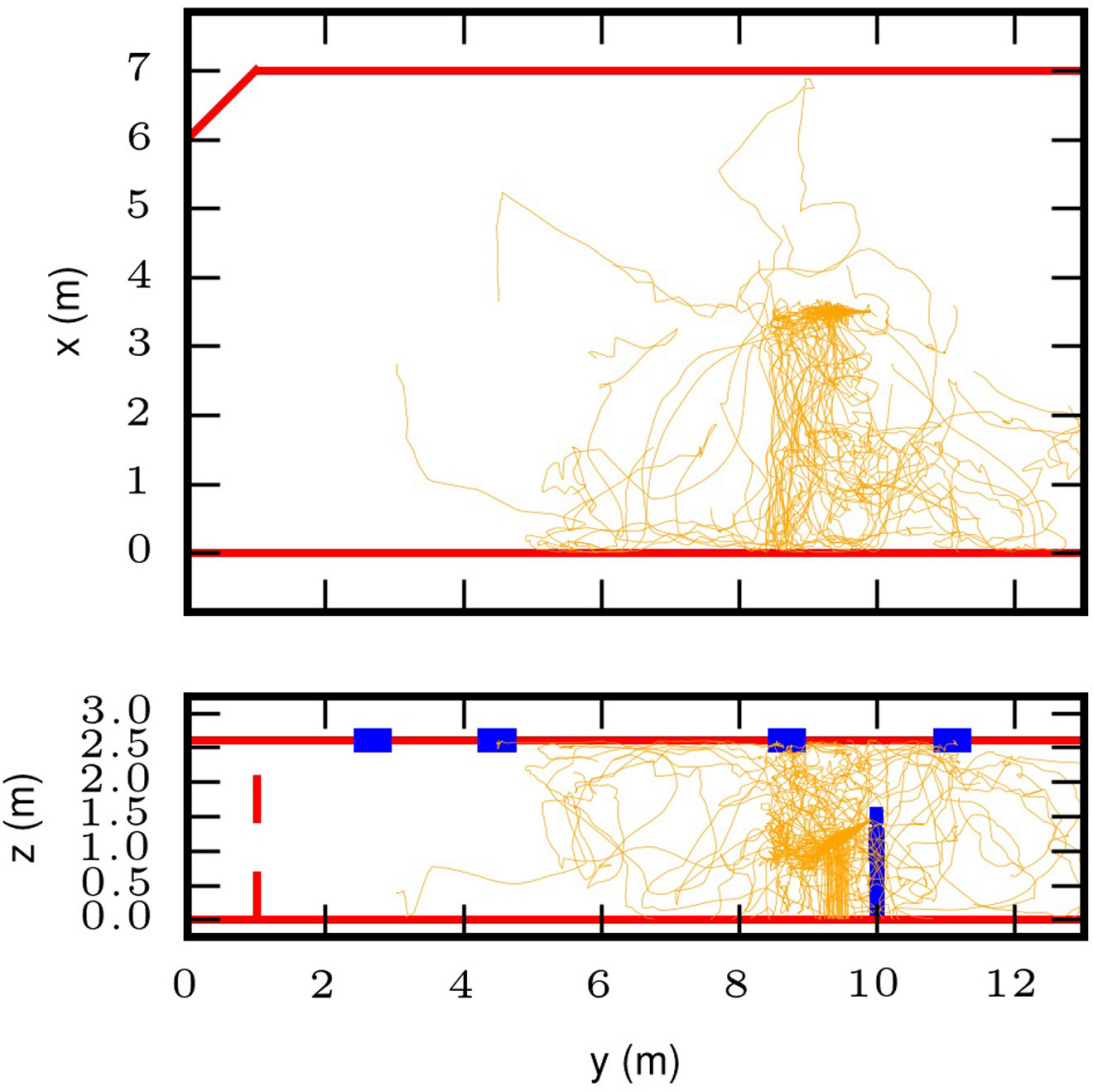
Droplet/particle tracks in plan view (upper) and side‐elevation (lower) view for the intermediate size bin (20 μm < d_0_ ≤ 100 μm) for the lowest evaporation case, cold and humid (T = 16°C and RH = 70%).

Figure 5 shows intermediate size droplets (20 μm < d_0_ ≤ 100 μm) for the lowest evaporation case, cold and humid (T = 16°C and RH = 70%). The asymmetric dispersion effect is evident here. Also, the side‐elevation shows how under the cold and humid conditions that the droplets are evaporating less and can be seen falling to the floor in front of the person.

**FIGURE 6:**
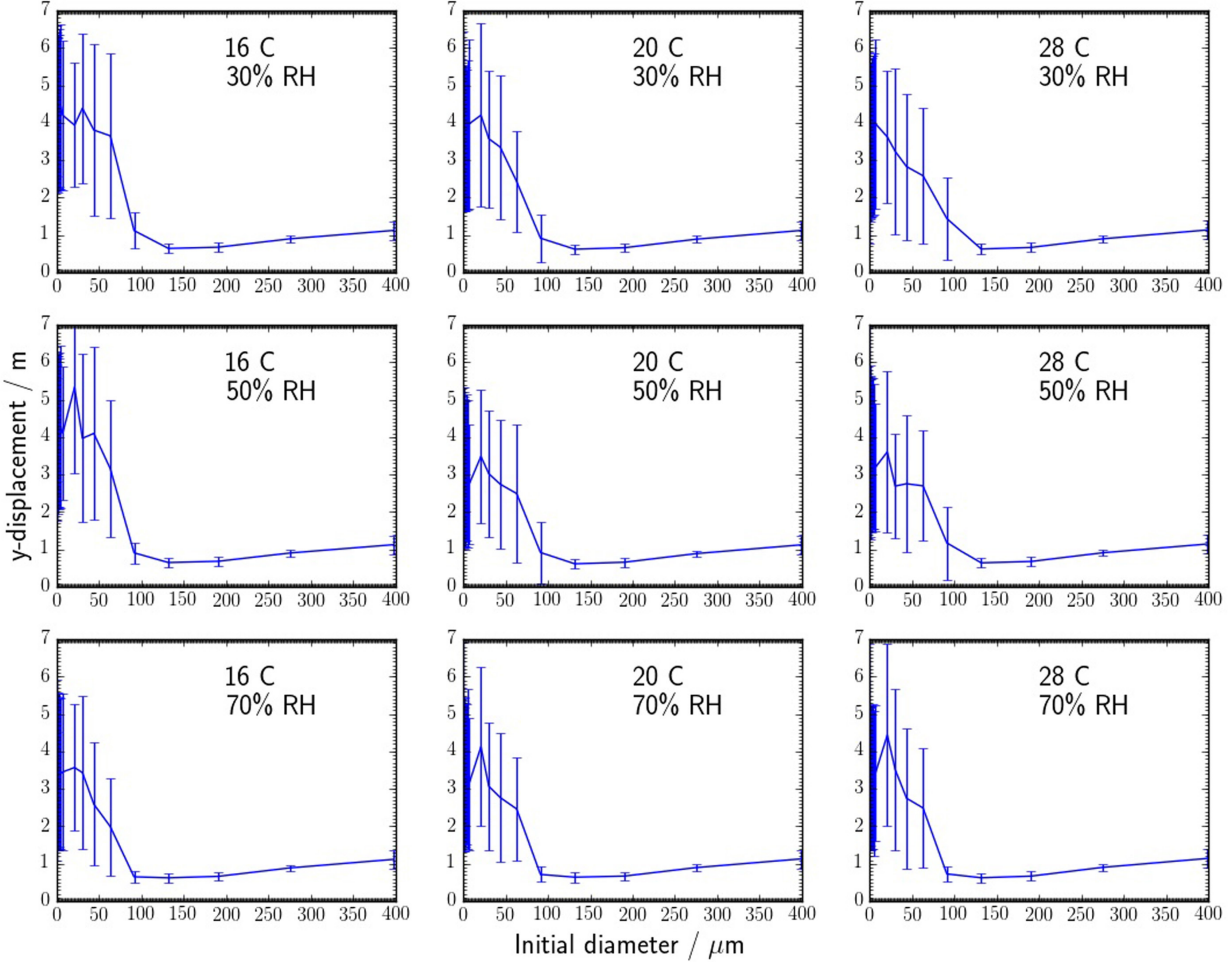
Distance traveled (before hitting the floor, walls or ceiling of the room) vs initial diameter, *d*_*0*_, plots for all temperature and RH conditions. The error bars show ± one standard deviation.

Graphs were produced (see Figure 6) to show how far the droplets/particles traveled in the direction of the cough (y‐axis) before hitting the floor, walls or ceiling of the room by the end of the simulation (5 min). The analysis does not differentiate between droplets/particles that have traveled in front of or behind the person. Data for all droplets/particles for each d_0_ size bin were combined.

It should be noted that all size bins did not contain the same number of droplets (see Supplementary Information S1 for the specific size bin diameters). Once a droplet has evaporated fully, the diameter of the resulting solid particle, *d*_*final*_, is 0.23 times *d*_*0*_. Xie et al.^3^ showed that at 50% RH, 80 μm droplets evaporate completely (they did not contain any non‐volatile components) in approximately 20 s and 50 μm droplets take 7.5 s.

Figure 6 shows that droplets/particles with an initial diameter of ≥132 μm on average only travel between approximately 0.5 and 1.2 m from the infected person, and a reasonable proportion of this distance will be spent at a height below the breathing zone of a standing person. It is therefore likely to be relatively rare that these droplets impact another person's mucous membranes. Droplets/particles with an initial diameter ≤ 63 μm (*d*_*final*_ ≤ 15 μm) travel on average 2 m or more (before hitting the floor). In some cases (16°C at both 30% and 50% RH) the average *d*_*0*_ = 63 μm droplet/particle traveled more than 3 m. It is difficult to separate the effect of temperature and RH from the general variability in these results, but the droplets/particles generally travel the shortest distance in the cold and humid case (16°C & 70% RH).

These data can be compared to data from the Xie et al.^3^ simple model of exhaled pure water droplets. In the Xie et al. model, at 20°C and RHs of 30%, 50% or 70%, droplets with *d*_*0*_ greater than approximately 80 μm traveled about 1.5 m before hitting the ground and the distance traveled was not affected by a change in RH.

In the CFD model here, the distance traveled is shorter, but this is likely due to the difference in the injection heights of the droplets (2 m in the Xie et al. model and 1.43 m in the CFD) and because the Xie et al. cough is a continuous horizontal jet (vs. an angled down puff in the CFD). The CFD also showed that a change in RH had very little effect on the larger sizes. The transition into the large droplet phase occurred at a slightly larger diameter in the CFD, approximately 91 μm in the CFD vs approximately 80 μm in the Xie et al. model. This could be because droplets with diameters less than 95, 80 or 65 μm (the exact size is dependent on the RH) evaporate before they reach the floor in the Xie et al. model.

In the Xie et al. model, the distance traveled peaks at between 2.0 and 2.5 m for 35 –45 μm droplets (depending on the RH). In the CFD, the distance traveled continues to increase as the initial diameter reduces, as these droplets cannot evaporate fully. The wide standard deviations in the CFD data are due to the complex room airflow and turbulence, which can reduce sedimentation, and are not included in the Xie et al. model.

The data above can be considered in relation to a susceptible person wearing a face covering. A simple cloth face covering may have a low efficiency for smaller particles (e.g., approx. 58% for sizes up to 6 μm according to Konda et al.^36^), with much higher efficiencies for the larger sizes (e.g., 94% for 100 μm to 1 mm according to Aydin et al.^37^).

A particle with *d*_*final*_ = 6 μm will have *d*_*0*_ = 26 μm and this could carry 25 viral RNA copies for the viral load assumed in the model (see Equation 1). Therefore, droplets with relatively large initial diameters, produced by a non‐mask‐wearing infectious person coughing, may evaporate to a size where they are not very effectively filtered by a cloth face covering worn by a susceptible person. In addition, the evaporation of these droplets, with relatively large initial diameters, means that they can often travel more than 4 m, even under cold and humid conditions.

### 3.3 Exposure

This section shows exposure to viral RNA calculated according to the method described in Section 2.5.1. Contour plots are shown initially to illustrate the spatial distribution of exposure and how it is affected by temperature and RH. Following this, the distribution of exposures within three 1 m^3^ volumes at increasing distances from the infected person (see Figure 9) under the different temperatures and RHs are shown. The data has then been analyzed using quantile regression to determine whether any changes due to distance from the person, temperature or RH are statistically significant.

#### 3.3.1 Contours

**FIGURE 7:**
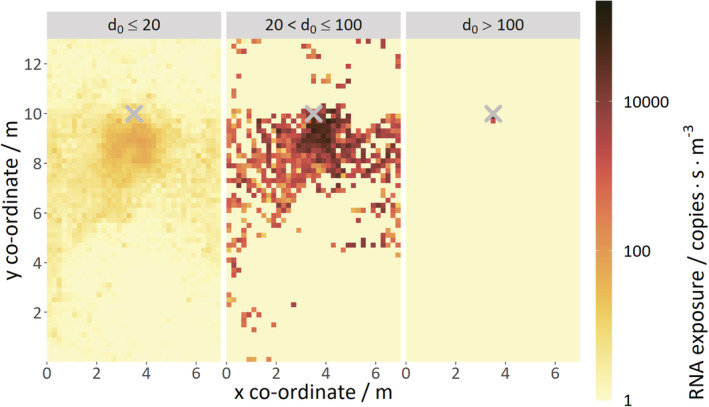
Contour plots of viral RNA exposure on a horizontal plane at 1.4 m; for three size bins; d_0_ < 20 μm (left), 20–100 μm (middle), >100 μm (right); at T = 28°C and RH = 30%. The ‘X' indicates the location of the coughing person. 1 RNA copy·s·m^−3^ was added to all the data to allow it to be plotted on a logarithmic scale.

**FIGURE 8:**
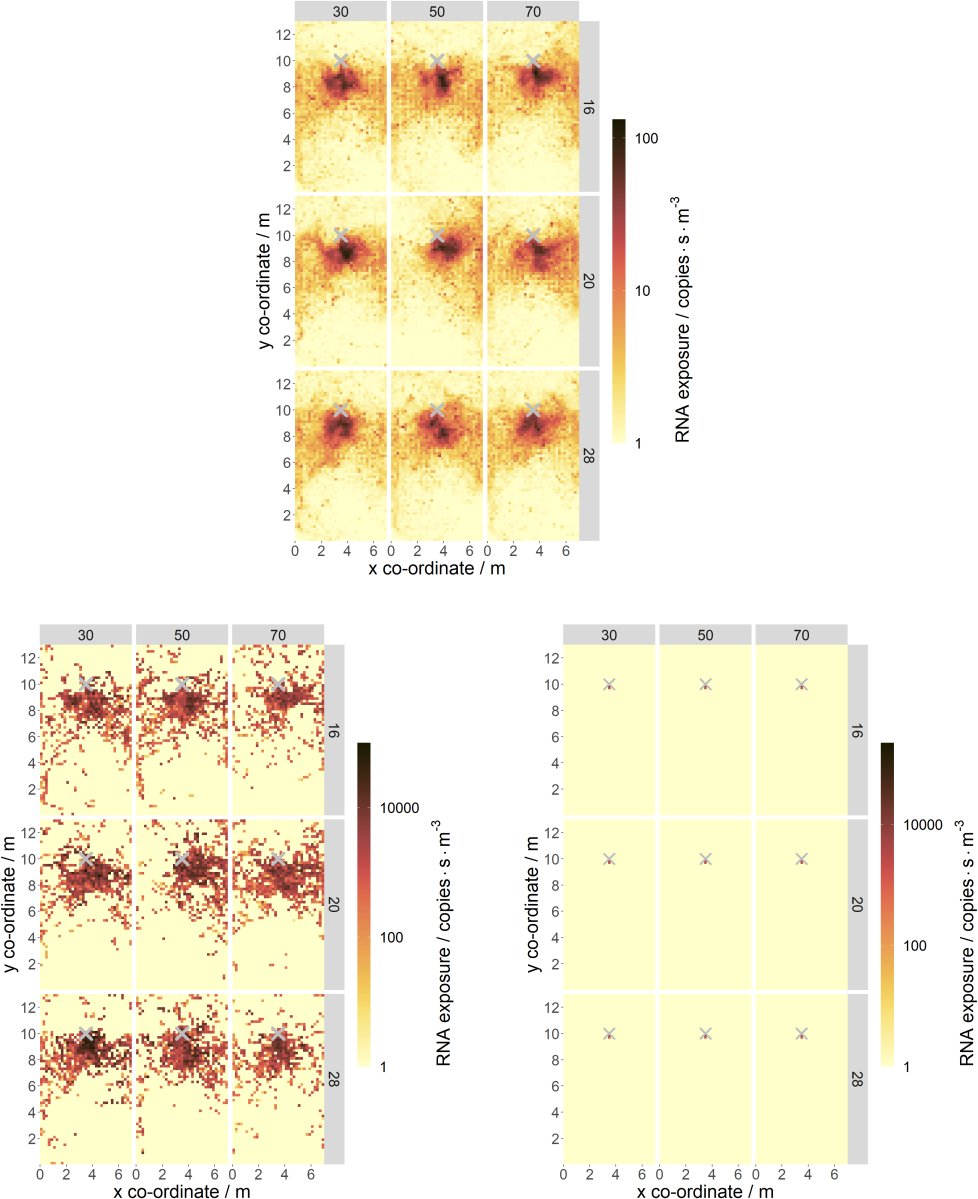
Contour plots of viral RNA exposure on a horizontal plane at 1.4 m, for all temperature (shown in the side gray bars in °C) and RH (shown in the top gray as a percentage) conditions. Three particle bins are shown: d_0_ ≤ 20 μm (upper), 20 μm < d_0_ ≤ 100 μm (lower left), d_0_ > 100 μm (lower right). The ‘X' indicates the location of the coughing person. The exposure scale is different for the d_0_ ≤ 20 μm image. 1 RNA copy·s·m^−3^ was added to all the data to allow it to be plotted on a logarithmic scale.

The contour plots in Figure 7 and Figure 8 show viral RNA exposures on horizontal planes spanning the meeting room at a height of 1.4 m (just below the mouth). The exposures were calculated within (0.2 m)^3^ sub‐volumes. Data is shown for the size bins used for the particle tracks previously. All plots show data averaged from the five coughs with 5 min of mixing time per cough.

**FIGURE 9:**
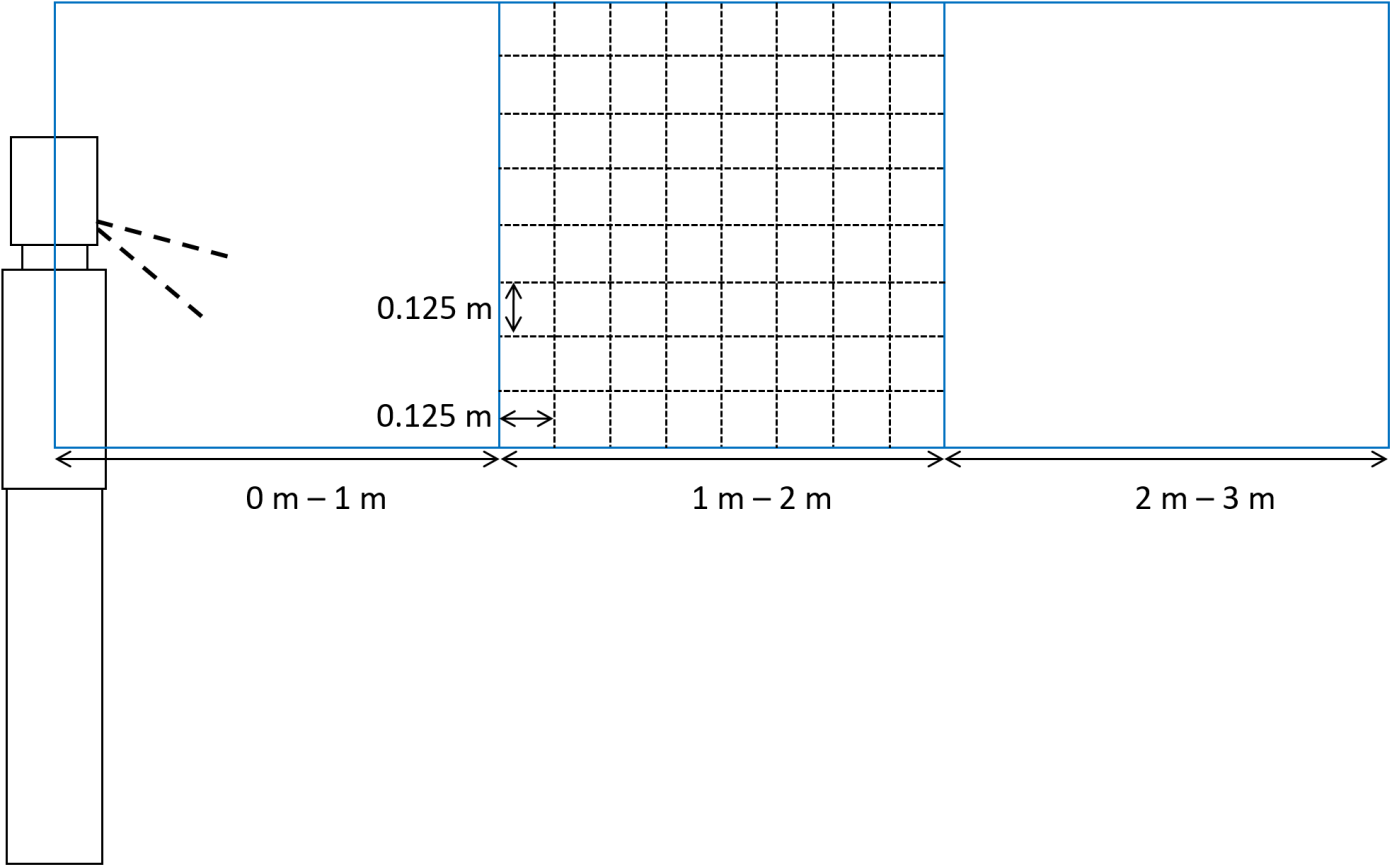
Layout of the three analysis volumes, with the sub‐volume shown for the middle volume. The infected person is shown to the left of the image with the angle of the exhaled jet indicated by thick dashed lines.

**FIGURE 10:**
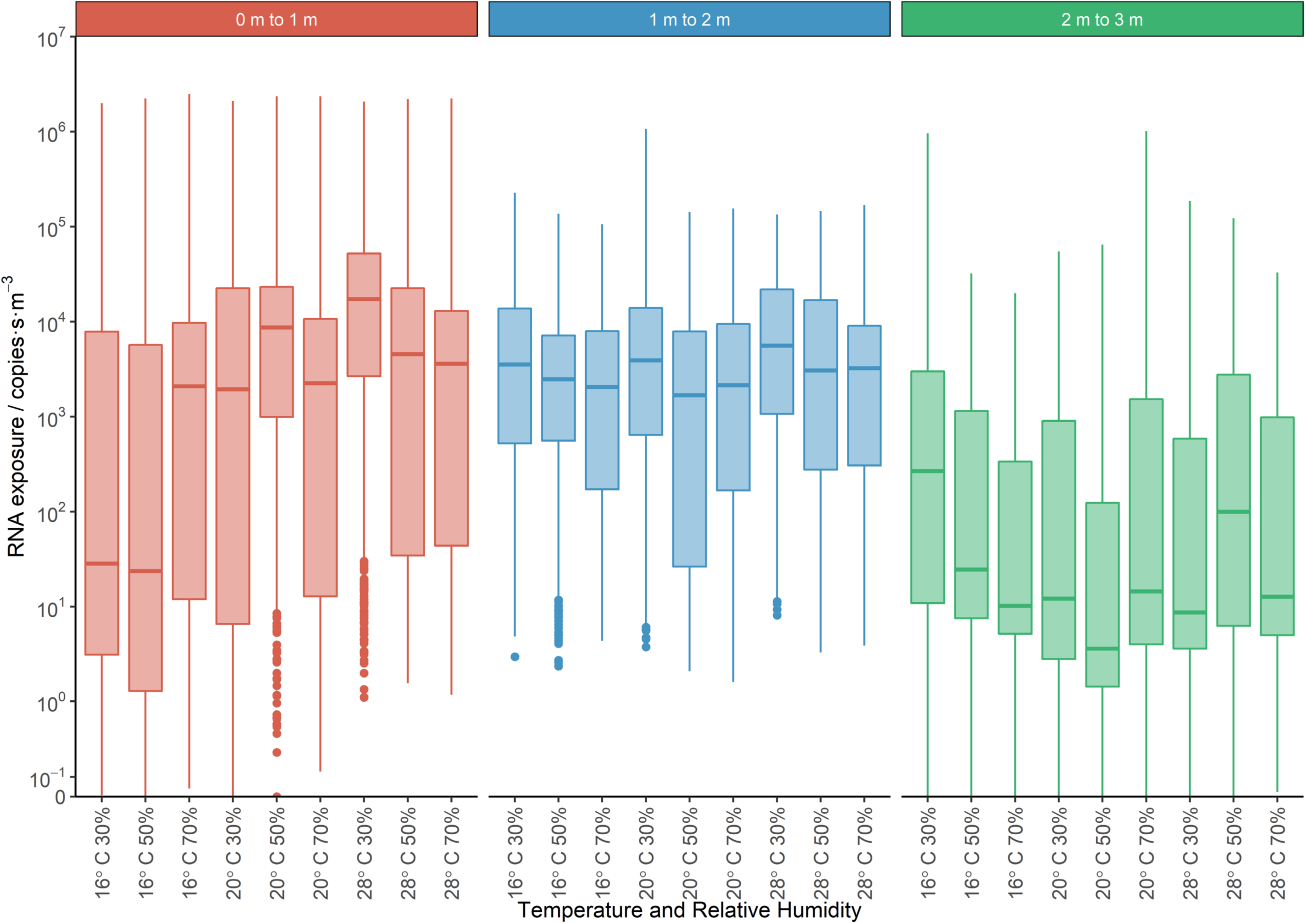
Box and whisker plots for exposure showing results for each simulation. Data is show for three breathing height analysis volumes at increasing distance from the person.

**FIGURE 11:**
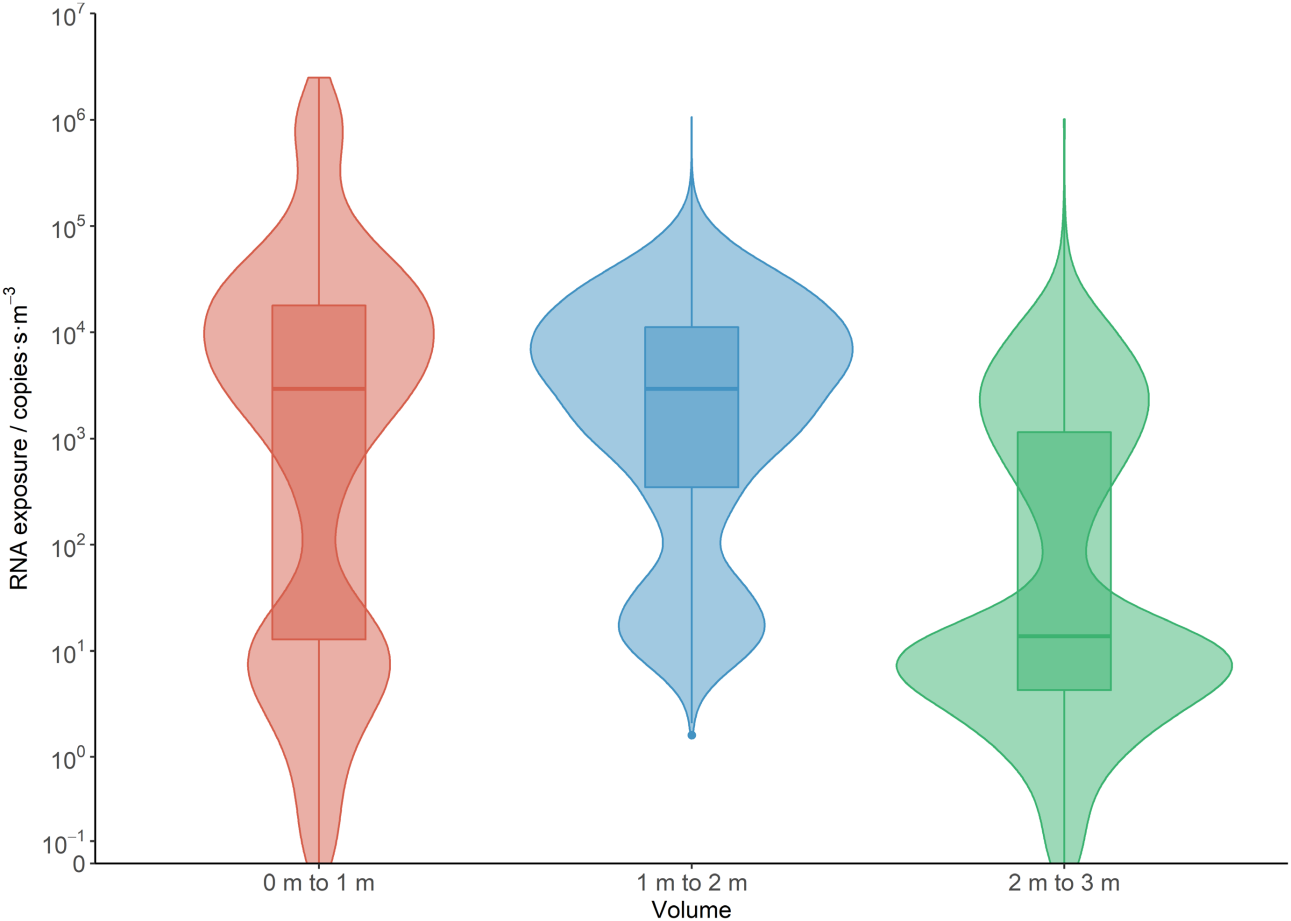
Violin plots (with added box and whisker) for exposure showing the effect of distance from the infected person. Data is shown for the three breathing height analysis volumes at increasing distance from the person.

**FIGURE 12:**
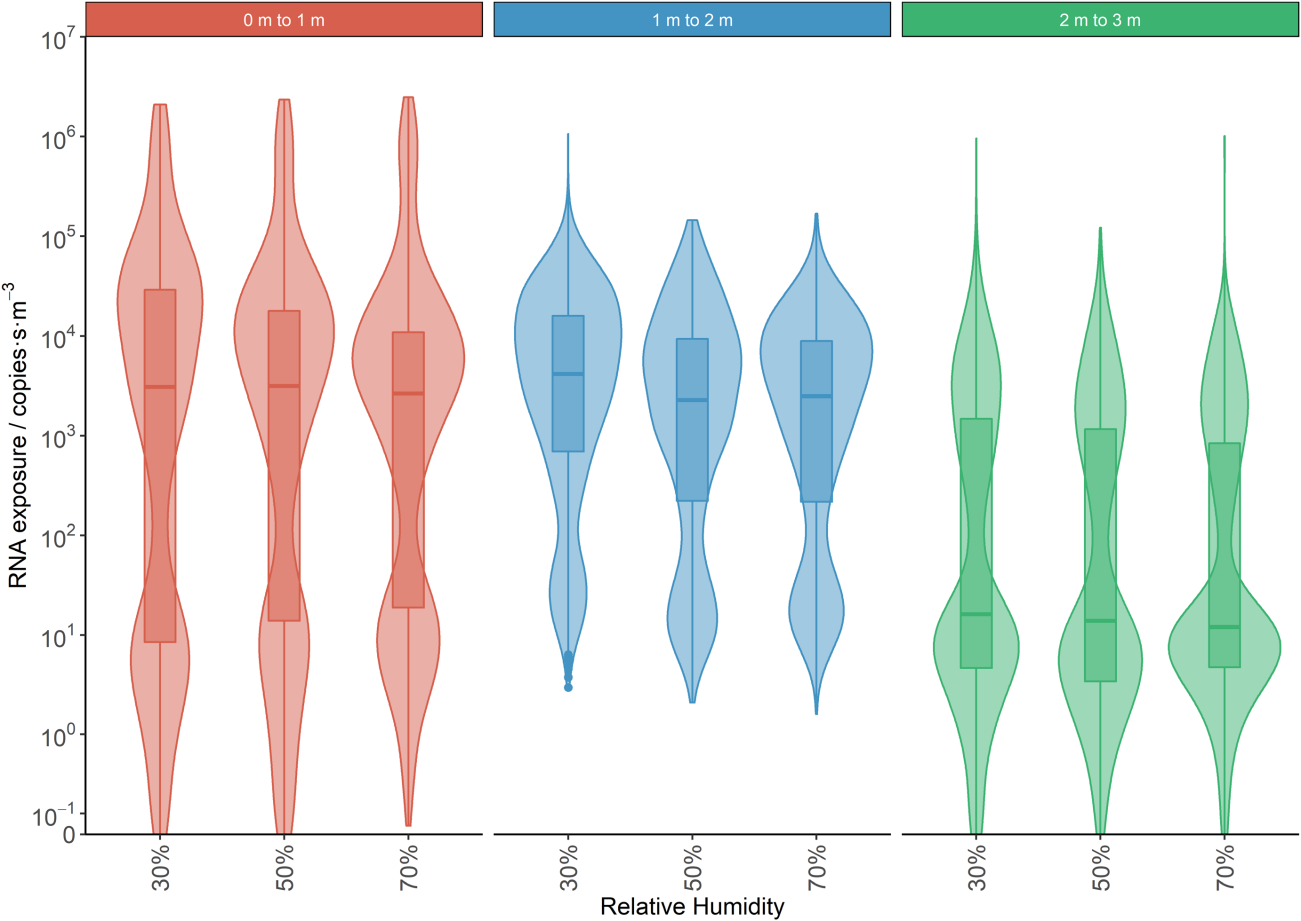
Violin plots (with added box and whisker) for exposure showing the effect of RH. Data is shown for the three breathing height analysis volumes at increasing distance from the person.

**FIGURE 13:**
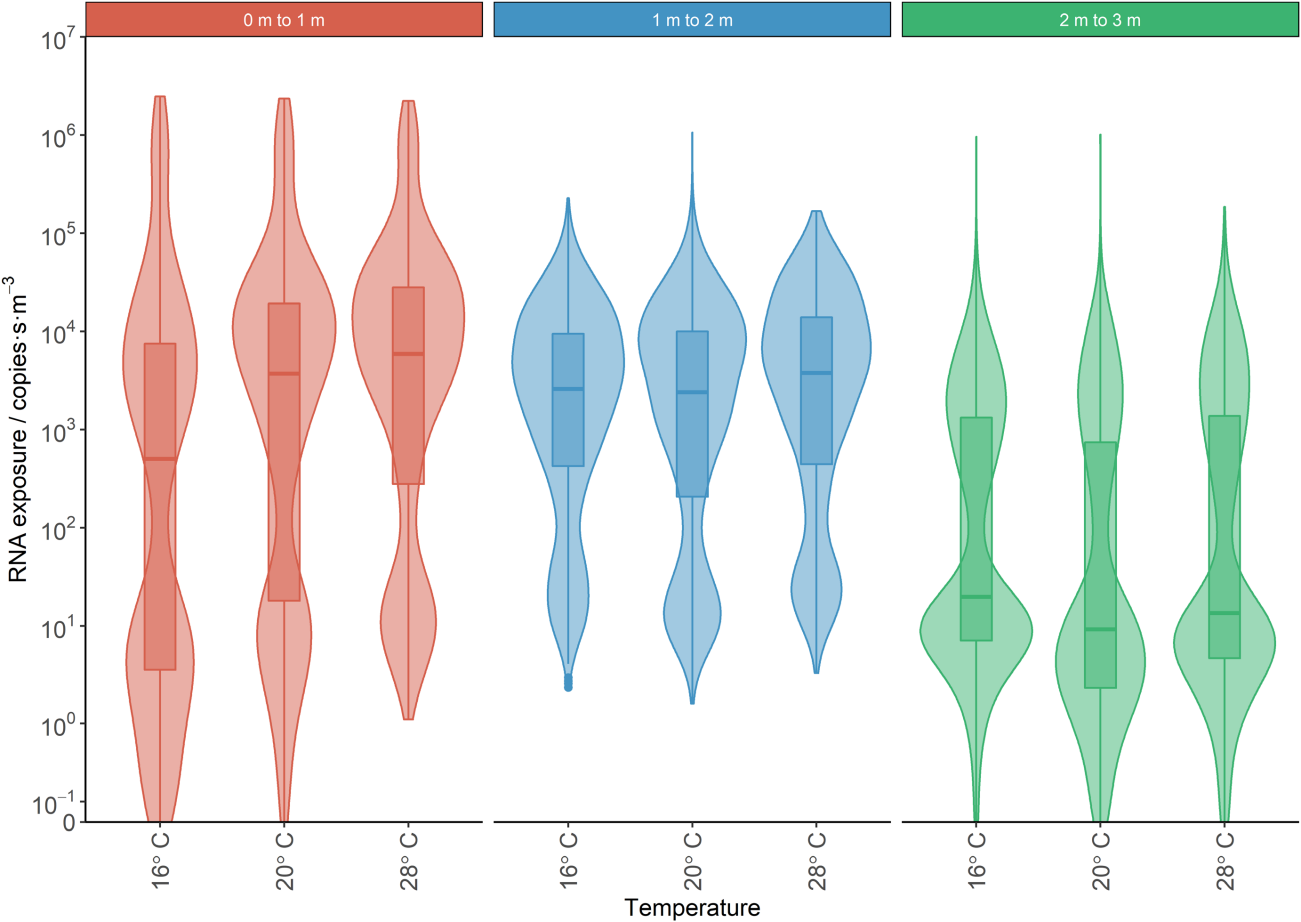
Violin plots (with added box and whisker) for exposure showing the effect of temperature. Data is shown for the three breathing height analysis volumes at increasing distance from the person.

Figure 7 shows how the exposure varies between the three size bins for the highest evaporation, hot and dry case (T = 28°C and RH = 30%) and that the bulk of the exposure is carried in the intermediate size bin. The largest droplets/particles carry the most virus but are too heavy to travel far from the infected person. The smallest droplets/particles are quite well dispersed around the room but carry little or no virus.

The exposures from the smallest droplets/particles are two or more orders of magnitude lower than those from the intermediate sizes. Intermediate sizes disperse a similar distance around the room as the smaller sizes, and exposures are still moderately high up to 3 m directly in front of the person. The high exposure region extends even further towards the side of the person due to the ventilation airflow in the room.

Some of the intermediate‐size droplets/particles travel to the far‐side of the room (still within the breathing zone) within the 5‐minute simulation, resulting in remote patches of high exposure. Therefore, for this mechanically ventilated room, mid‐size droplets/particles carrying large numbers of viruses have been shown to create a hazard region, which extends beyond the typical 2 m social distance spacing due to the airflows within the room.

If it was practical to average data across more coughs, it is expected that the exposure contours would remain approximately the same shape, but the contour patterns would be smoother than illustrated in Figure 7.

Figure 8 shows the effect that temperature and RH have on the spatial distribution of exposure. The largest size droplets/particles are not affected significantly by a change in temperature and RH when considering the whole room scale.

Due to the unsteadiness of the airflow in the room and the resulting variability in the paths that the particles follow, as discussed in Section 3.2, it is not clear whether temperature and RH have much effect on the exposures from intermediate or small‐size droplets/particles. More coughs would be required to show whether any effect is statistically significant or not.

#### 3.3.2 Distribution of possible exposures

To highlight whether there are significant changes in the exposure (over 5 min) as a function of temperature and RH, the viral exposure was calculated within three 1 m^3^ volumes at increasing distances from the person. The volumes were centred vertically and laterally on the mouth, so covered a height from 0.93 to 1.93 m. The positioning was chosen to represent the regions in which the mouth of a susceptible person may be located. The exposure was calculated in (0.125 m)^3^ sub‐volumes as described in Section 2.5.1.

Figure 10 shows box and whisker plots for all calculated exposure across the three analysis volumes for all simulations. The box extends from the first to the third quartile, so that the length of the box is in the interquartile range. The upper whisker extends up from the top of the box to the third quartile plus 1.5 times the interquartile range, bounded by the maximum data point. A very small number of the sub‐volumes had no droplets/particles pass through them, so have an exposure of zero. The median and interquartile range values for these figures are given in the Supplementary Information S1.

Figure 10 shows that there is a large range of possible exposures within each volume for each simulation (greater than six orders of magnitude in the 0–1 m volume). Therefore depending on where the receptor person is located, they could receive a high or low dose. In the 2–3 m box, there is an overall decrease in exposure compared to the other volumes because less of the exhaled droplets reach this volume.

The exposure distributions in the first volume are wide because the coughed jet only passes through the bottom part of this region. This means there are sub‐volumes with higher exposures and sub‐volumes with lower exposures. All the exhaled droplets pass through the bottom part of the volume but do so quickly, therefore they do not contribute as much to the exposure as might be expected. By the second volume, the jet will have slowed and droplets will have reduced in size. This means that the droplets will become more strongly affected by the room airflows. As a result, they mix more uniformly, and more slowly, across the second volume, so medians remain high and the distributions are tighter. None of the largest droplets from the exhaled jet reach the third volume, so the exposure here is made up of the smaller and intermediate sizes, which remain airborne and recirculate around the room.

The exposure distributions within each 1 m^3^ volume were bimodal and this is possibly a reflection of the unsteady airflow in the room. The higher exposure mode may represent the cases where the exhaled droplets are mixed symmetrically and the lower mode where flow is asymmetric and a number of the droplets miss a large number of the sub‐analysis volumes. Violin plots are used in subsequent graphs to show the shape of the exposure distributions.

A series of regression models were used to determine whether there were statistically significant differences between the variables of interest, including any possible interactions. Due to the nature of the data distributions, the RNA exposure was first transformed on a natural log scale. However, no sensible transformation of these data enabled a suitable linear regression model to be fitted. As a result, quantile regression models were fitted to the log RNA exposure.

All analysis was carried out in R Studio (V1.2.1335). Quantile regression models were created using the rq function from the quantreg package on the median log RNA exposure.

Analysis was initially carried out using the combined data from all three analysis volumes. However, a number of statistically significant interactions were found in the model selection process, highlighting the interdependency between temperature and RH, which was also found to vary between volumes. Additional quantile regression models were therefore formulated for each of the volume subsets, to better understand the interactions (see Supplementary Information S1 for the results of the chosen quantile regression models).

To highlight the effects of distance from the infected person, RH and temperature, data for these three variables is shown in Figures 11, 12, 13. These figures show violin plots overlaid on box and whisker plots. The violins have been used as they show the shape of the exposure distributions; they show the relative probability of a particular exposure being recorded in a sub‐volume. In Figure 11 the data from all nine simulations have been combined in each analysis volume. In Figure 12, the data for all temperatures are combined to show the effect of RH and in Figure 13 the data from all RHs are combined to show the effect of temperature. The median and interquartile range values for these figures are given in the Supplementary Information S1.

Overall, a statistically significant decrease in exposure is found as the distance from the infected person is increased from 0–1 to 2–3 m (but not from 0–1 to 1–2 m) and as the RH is increased.

A statistically significant increase is observed as the temperature is increased from 16 to 28°C, but not from 16 to 20°C. However, when exploring interactions between covariates, it is clearly evident that the direction of change and magnitude of log RNA exposure is dependent upon the volume, temperature and RH (see Figure 10). This highlights the need to further explore the effect of temperature and RH by volume.

The overall difference by volume is illustrated in Figure 11, in which the median exposure reduces from 2940 to 2938 copies·s·m^−3^ when moving from the 0–1 m volume to the 1–2 m volume; representing a very small change. However, there is a marked decrease in the 2–3 m volume, where the median is just 13.8 copies·s·m^−3^, a factor of greater than 200 reductions compared to the 0–1 and 1–2 m volumes. A similar effect is shown in Figure 7, but the large drop in exposure between the 1–2 and 2–3 m volumes is not so apparent on this 1.4 m high plane. The changes in median exposure should also be considered in relation to the width of the distribution, that is, greater than six orders of magnitude in some cases.

There is a change in the shape of the distributions from the 0–1 m volume to the 1–2 m volume. So, even though the change in the median exposure is small, someone in the first volume is more likely to receive a high exposure (e.g., greater than 10^5^ copies·s·m^−3^) than they are in the second volume.

This shows that, for two people standing face‐to‐face, 1 m social distancing would not have that much effect on the median exposure in absolute terms. The social distancing of 2 m or more would dramatically reduce the chance of receiving a high exposure from a coughing person. However, due to the unsteady airflow in the room and the stochastic nature of the particle transport, there are still potentially some locations within the 2–3 m volume where exposures are almost as high as the highest exposure in the nearer volumes (as also indicated in Figure 7). Out of all nine simulations, less than 0.1% of locations (3/(9 × 512)) in the 2–3 m volume had greater than 10% of the average highest exposure recorded in the 0–1 m volume, that is, greater than 2.2 × 10^5^ copies·s·m^−3^.

As stated earlier, people standing very close to the infected person could receive much higher exposures than indicated by the data shown here. This is because the exposure reported is a function of the sub‐volume size when the droplets or particles are not uniformly distributed within the sub‐volumes.

The overall difference by RH is illustrated in Figure 12. In the 0–1 m analysis, volume the median exposure reduced from 3095 to 2647 copies·s·m^−3^ as the RH increased from 30% to 70%. Similarly, in the 1–2 m analysis volume, the reduction in the median exposure was 4179–2488 copies·s·m^−3^, for the same increase in RH.

In addition, in the 1–2 m analysis volume, RH was considered an important factor to control for in the model and the reduction in log RNA exposure from 30% to both 50% and 70% RH was statistically significant. However, in the 2–3 m analysis volume, there was minimal absolute change in the median exposure although the change from 30% to 70% RH (16–12 copies·s·m^−3^) was statistically significant.

The changes in median exposure due to RH in the 0–1 and 1–2 m volumes are larger than the reduction in exposure when moving from the 0 to 1 m volume to the 1–2 m volume. However, the reduction in exposure when moving from the 1–2 and 2–3 m volumes is much greater than any changes due to RH.

The overall difference according to temperature is shown in Figure 13. Although a statistically significant increase is observed as the temperature increases from 16 to 28°C overall, the magnitude and direction of this change vary between volumes. For example, in the 0–1 and 1–2 m volumes, the increase to 28°C is statistically significant. However, for the 2–3 m analysis volume data, compared to a temperature of 16°C, there was actually a statistically significant decrease in exposure at both 20 and 28°C.

In the 0–1 m volume, the median exposure increases more than ten times (504–5890 copies·s·m^−3^) from 16 to 28°C. In the 1–2 m volume, the increase is much smaller, 2602–3789 copies·s·m^−3^. In the 2–3 m volume, the median exposure decreased from 19.8–13.5 copies·s·m^−3^.

It is not clear whether the large increase in the median exposure in the 0–1 m volume, as temperature increases, is a true reflection of the size of the temperature‐driven effect. Figure 10 shows that the median exposures are low (less than 100 copies·s·m^−3^) for 16°C at both 30% and 50% RH, but the median is high (greater than 1000 copies·s·m^−3^) for 16°C at 70% RH. Due to the bimodal shape of the exposure distributions, a small change in the distribution can result in the median jumping from the top mode to the bottom mode.

### 3.4 General discussion

The results from our CFD modeling support the finding of most relevant previous studies^1^, ^3^, ^4^, ^5^, ^6^, ^10^ that large droplets under dry conditions evaporate quickly, stay suspended in the air and therefore present a hazard for longer, that is, a higher exposure. The data supports the finding of Chen,^5^ that an increase in the temperature does not always result in an increased exposure. The findings here are not as clear‐cut as those of Chen. However, the Chen model was for a stationary single droplet only.

The CFD modeling by Li et al.^6^ of droplet transport in an unventilated room showed that a change in RH was more important than a change in temperature when it came to evaporation. A similar result has been shown here, that the change in exposure due to a change in RH is always statistically significant, but is not always statistically significant for a change in temperature.

It is likely that the effect of temperature and RH would be increased if a wider range of values were considered. The ranges chosen for this study were based on those likely to be seen in an air‐conditioned office space.

Only coughing has been considered in the current models and the direction of the cough (i.e., the exhaled jet of air) is below the horizontal.^27^ If the coughed jet was horizontal or if the individual was sneezing, talking or breathing, the results may be different. However, it is expected that the basic influence of temperature and RH driven on the evaporation rate would be the same, so the overall effect on exposure would likely be similar.

For sneezing, there may be considerably more droplets^38^ and the force of the sneeze may project these droplets further into the room.^39^ This would have a significant effect on the magnitude of the exposure and possibly a small effect on the spatial distribution of exposure. Conversely, for breathing or quiet talking, the number of droplets and their initial momentum would be lower. Therefore, this is likely to reduce the magnitude of exposure as well as have some influence on the spatial distribution. As well as different types of exhalation, it would be interesting to study the effect of wearing a face covering. These may remove a high proportion of the larger droplets/particles, that is, those that are most affected by RH, so the overall effect of temperature and RH on exposure could be less than shown here for the no face covering case.

As the model was based on a real room, only one air change rate has been considered, along with one supply/extract vent layout and one location of the infected person relative to the vents. An increase in the air change rate will increase the turbulence and mixing in the room so will likely reduce any effect from temperature and RH. The opposite is likely to be true for a decrease in the air change rate.

The droplets used in the present work had a vapor pressure of pure water so will evaporate more quickly than saliva or saline droplets (the reasons for using the vapor pressure of pure water are discussed in Section 2.2.1). The slower the evaporation the smaller the effect that temperature and RH will have. Therefore, if these models were rerun with more realistic multicomponent droplets, it would be expected that any effect from a change in temperature and RH would be reduced.

The results are expected to depend, to some extent, on the droplet size distribution used to represent what is exhaled during a cough. The BLO distribution was used for this study, so it would be interesting to repeat the work with different size distributions, particularly those such as Duguid,^38^ which has a higher proportion of droplets in the mid‐size range. If the proportion of droplets in the mid‐size range was increased, it is possible that the effect of temperature and RH would be enhanced, and the opposite would be true if the proportion of droplets in mid‐size range was decreased.

The effect of temperature and RH on the deposited viral concentration has not been reported here but it would be interesting to see if the effect corresponds with those for exposure. If a low RH results in higher exposures, it would be expected that the same condition would result in lower deposited concentrations. A similar counter‐effect was shown by Parhizkar et al.^10^ Therefore, any temperature or RH control to limit the airborne exposure is likely to increase the deposited hazard. If this deposited material is mainly on the floor of the room then this might be acceptable. However, if the deposition is on surfaces such as desks, then it might be necessary to implement measures to reduce fomite transmission through other means, for example, more regular surface cleaning, hand sanitizing, mask wearing and even behavior change (less face touching). Indeed, a recent modeling study suggests that risks of fomite exposure could be higher in warmer and more humid environments due to both higher hand transfer efficiencies and longer virus survival.^40^

As stated in the introduction, only the fluid dynamics effects from a change in temperature and RH have been considered here. The decay in the viability of the virus in droplets was not included. While some of these effects could be included in a CFD model, it would be more effective to include others in a higher‐level model, such as a quantitative microbiological risk assessment model.^41^

## 4 CONCLUSIONS

A series of RANS CFD models have been developed to predict the effect of temperature and RH on the airborne exposure to SARS‐CoV‐2 from a coughing person in a mechanically ventilated meeting room or office space.

The novelty of this work is the coupling of spatial distributions of viral exposures and statistical analysis to evaluate viral exposure due to different size exhaled droplets. The analysis was used to indicate whether the effects of temperature, RH and distance from the infected person have a statistically significant effect on the airborne exposure. The modeling demonstrates the importance of evaporation on exposure to respiratory aerosols and droplets. The results demonstrate that evaporation leads to mid‐sized droplets reaching a size where they can often remain airborne over distances of more than 4 m. For a case where viral load [RNA copies·m^−3^] is independent of the initial droplet size, evaporation could result in exposure to particles that are less than or equal to 6 μm in diameter, which would not be mitigated through simple face masks, and could carry much higher amounts of the virus than the final particle size in the air suggests.

In the mechanically ventilated room studied, with all the associated complex air movement and turbulence, increasing the RH resulted in a statistically significant reduction in the exposure. However, this effect may be so small in absolute terms that other factors, such as moving closer or to the side of the person or fluctuations in the airflows, could rapidly counter the effect.

The effect of temperature on the exposure was more complex, with a positive correlation shown up to 2 m from the infected person, but a negative correlation in a region from 2 to 3 m. In all instances, moving away from the infected person resulted in a decrease in exposure, but the reduction in the median exposure was very small when moving from a region located 0–1 m in front of the person compared to a region located 1–2 m from the person.

If no other parameters are important, then setting the RH in a mechanically ventilated room to a high but comfortable level may slightly reduce the inhaled dose. If the RH is increased to reduce airborne exposure, then consideration should be given to the increase in the probability of fomite transmission. The effect of temperature on the exposure was more complex, with both positive and negative correlations. Therefore, within the range of conditions studied here, there is no clear guidance on how the temperature should be controlled to reduce exposure. The effect of distance shows that applying social distancing of 2 m would generally reduce the likelihood of two people, standing face‐to‐face, receiving a high exposure, however, results suggest that in some cases this will not be sufficient to mitigate the highest concentration of virus. The ventilation rate should not be compromised to achieve a high RH or low temperature.

## AUTHOR CONTRIBUTIONS

Timothy Foat: Conceptualization, Methodology, Validation, Visualization, Writing – Original Draft Preparation. Benjamin Higgins: Formal Analysis, Investigation, Software, Validation, Visualization, Writing – Review & Editing. Suzie Abbs: Formal Analysis, Methodology, Software, Visualization, Writing – Review & Editing. Thomas Maishman: Formal Analysis, Methodology, Visualization, Writing – Review & Editing. Simon Coldrick: Methodology, Validation, Writing – Review & Editing. Adrian Kelsey: Methodology, Software, Writing – Review & Editing. Matthew Ivings: Conceptualization, Supervision, Writing – Review & Editing. Simon Parker: Conceptualization, Methodology, Supervision, Writing – Review & Editing. Catherine Noakes: Conceptualization, Supervision, Writing – Review & Editing.

## CONFLICT OF INTEREST

No conflict of interest declared.

## Supporting information


**Appendix S1:** Supporting Information

## Data Availability

The data that support the findings of this study are available from the corresponding author upon reasonable request.
