# Supplementary material for: Modeling the effect of temperature and relative humidity on exposure to SARS‐CoV‐2 in a mechanically ventilated room
Source: Indoor Air. 2022 Nov 27;32(11):e13146. doi: 10.1111/ina.13146 (PMC10099484; doi:10.1111/ina.13146)
Supplement: Supplementary file 1 — Appendix S1: Supporting Information [file INA-32-0-s001.docx]

Supplementary Information

**The effect of temperature and relative humidity on exposure to SARS-CoV-2 in a mechanically ventilated room**

Timothy G. Foat, Benjamin Higgins, Charlotte Abbs, Thomas Maishman, Simon Coldrick, Adrian Kelsey, Matthew J. Ivings, Simon T. Parker, Catherine J. Noakes

1. **Mixing period following each cough**

The CFD simulation consisted of a cough followed by 5 min of mixing. A model was run to see whether this mixing period was sufficiently long to capture the bulk of the exposure for a person standing close to the infected person. This was done by calculating how long it took for the exposure within each of the (0.125 m)^3^ sub-volumes to reach 90% of their exposure at 30 min.

The results (Figure S1) show that approximately 50%, 69% and 88% of the sub-volumes, in the 0 m – 1 m, 1 m – 2 m and 2 m – 3 m volumes respectively, reached 90% of their 30 min exposure after 5 min. All three volumes reached approximately 38% within 1 min. The initial rapid increase in the percentages was due to the coughed jet travelling through the three volumes. The subsequent slower rise is from the droplets/particles being mixed through the volumes by the airflow in the room. As the jet has more momentum and the droplets are larger in the 0 m – 1 m volume, the droplets are less affected by the room airflows at there. It is not until the droplets reach the second or third volumes that they start mixing more effectively into the room. This is why the line for the 0 m – 1 m volume has the slowest increase after 1 min.


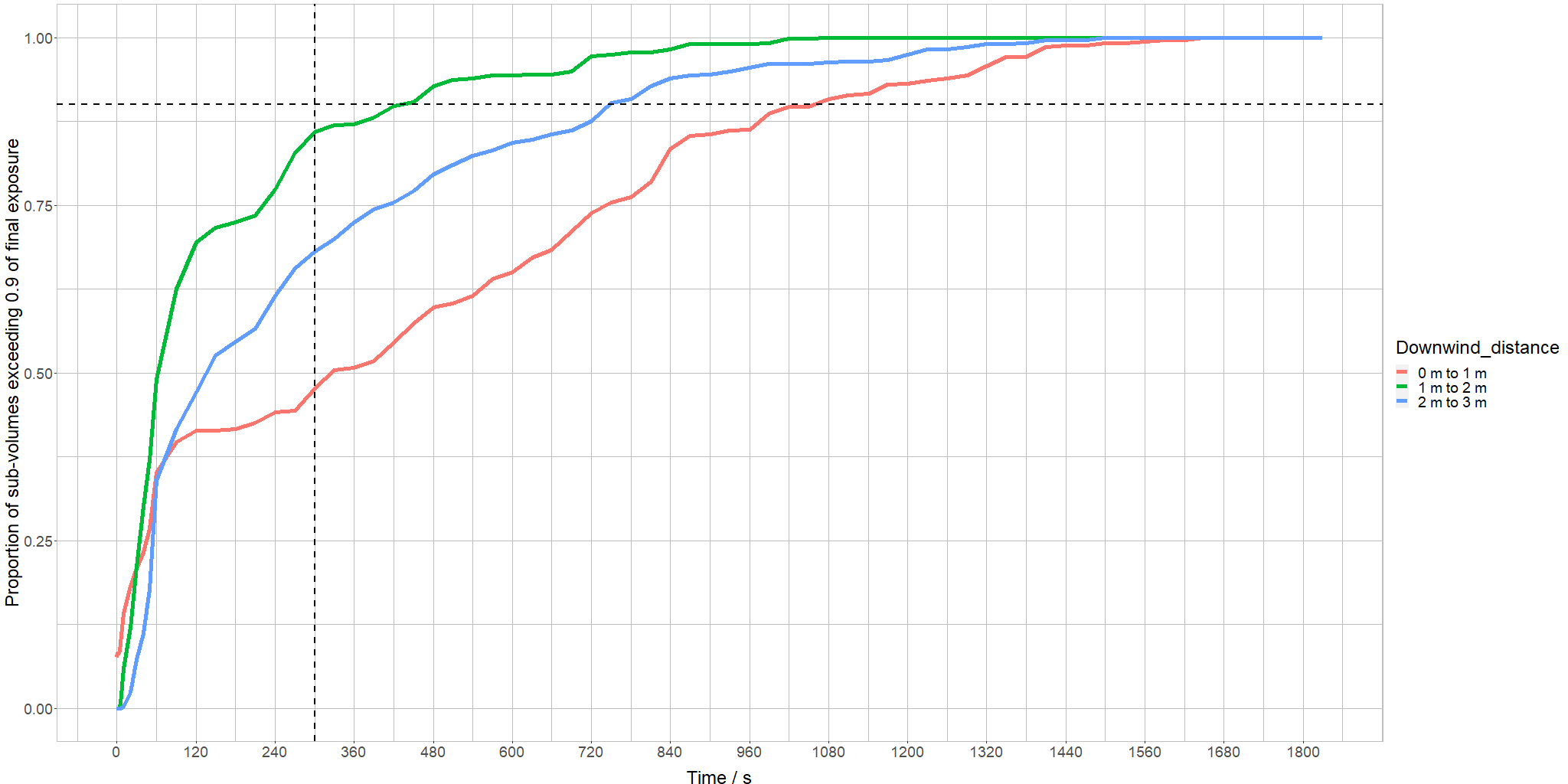


Figure S1 Graph showing how the proportion of the sub-volumes that have reached 90% of their 30 min exposure value changes with time. Lines are shown for the three 1 m^3^ analysis volumes. The dashed lines indicate 5 min and 90%.

1. **Viral load**

The SARS-CoV-2 viral load value used in this study was based on a value of 1.38 x 10^8^ copies·mL^-3^ from Singanayagam et al.^1^. This is the mean peak viral load for 50-year-olds and had 95% credible intervals of 8.91 x 10^7^ copies·mL^-3^ to 2.09 x 10^8^ copies·mL^-3^. The data included pre-alpha, alpha and delta variants and vaccinated and unvaccinated individuals. It is believed that this figure is for the concentration in the viral transport media rather than viral load in the respiratory tract. Chen et al.^2^ converted viral transport media concentrations to a respiratory viral load by using the transport media volume and the swab uptake volume. Chen et al. used a swab uptake volume of 0.128 mL, but a more reasonable figure of 0.05 mL is given by Roque et al.^3^. Singanayagam et al. do not appear to provide their transport media volume, so a value of 1 mL has been assumed, based on Chen et al. ^2^. This gives a dilution factor of 20, which can be compared to the dilution factor of 40 to 60, given by Roque et al. The resulting viral load is 2.76 x 10^9^ copies·mL^-3^ (2.76 x 10^15^ copies·m^-3^).

The Singanayagam et al. data shows that viral load changed significantly during the course of the infection and was dependent on the age of the person. It is also recognised that viral load may not be constant across the range of droplets sizes and may vary for different variants of SARS-CoV-2. Although, Singanayagam et al. reported no differences in the viral load across the variants they included in their study.

The viral load figure used here (2.76 x 10^9^ copies·mL^-3^) is equivalent to the 94th percentile in the Chen et al. ^2^ distribution of respiratory viral loads for SARS-CoV-2, so is representative of a reasonably high viral load.

1. **Model sensitivities**
   1. **Mesh sensitivity**

CFD models at three temperature and RH conditions were run with three different meshes to check for mesh sensitivity. The three conditions represented the lowest evaporation case (cold and humid), the highest evaporation case (hot and dry) and the middle case. The mesh was coarsened by increasing all cell sizes by a factor of 1.25 and was refined by reducing all sizes by a factor of 0.5. The results of the mesh dependency study are shown in Figure S2.


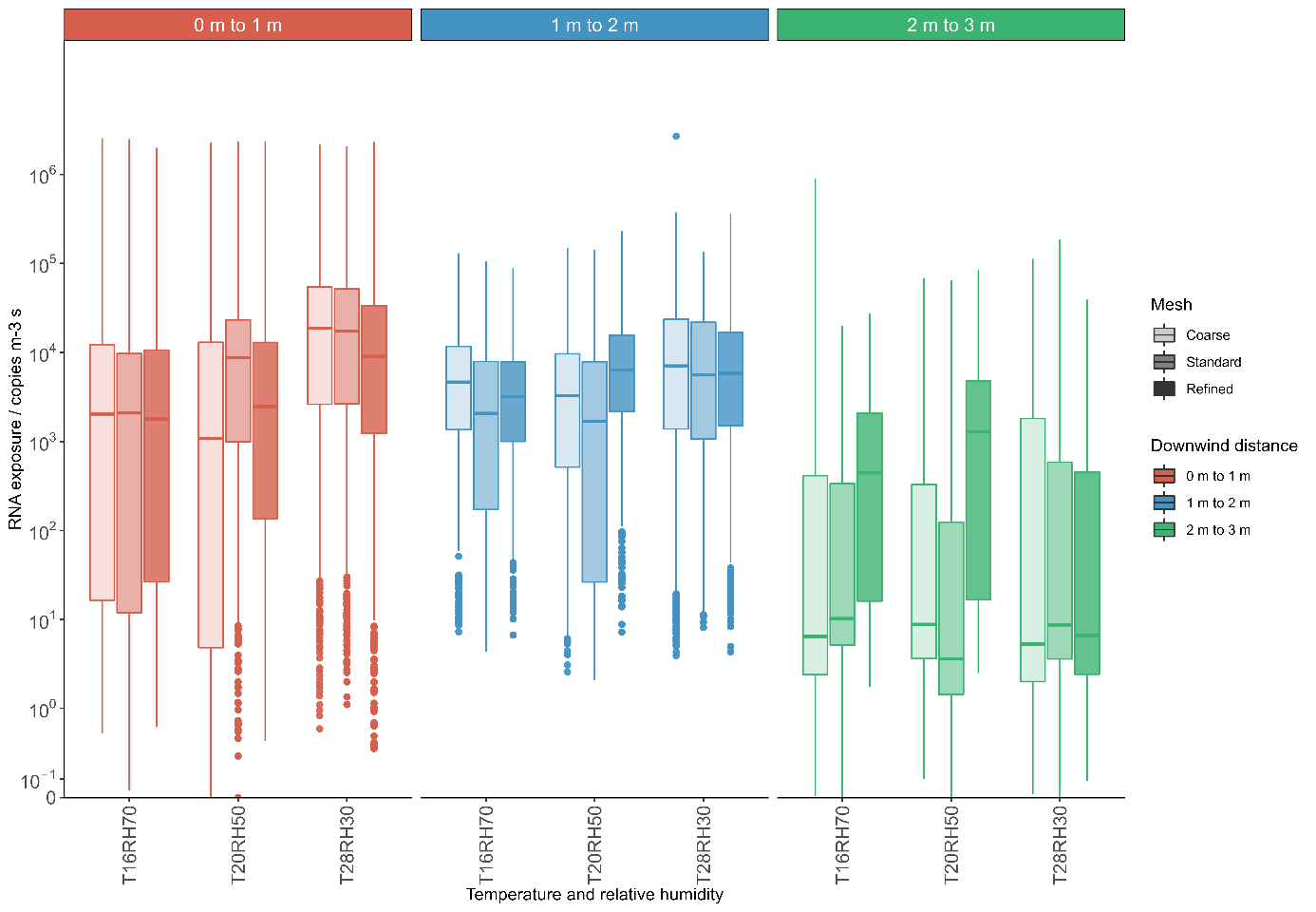


Figure S2 Mesh dependency box and whisker plots for exposure showing results for the coarse standard and refined mesh. Data is shown for three analysis volumes at increasing distance from the person. The baseline mesh is marked as standard in this graph. Temperatures are shown in Kelvin.

The results show that there clearly is some mesh dependency, but there are no overall trends in the data as the mesh is refined. The model appears to be less sensitive to a mesh refinement in the 0 m - 1 m and 1 m - 2 m volumes, and more sensitive in the 2 m - 3 m volume. Due to the unsteadiness in the airflow in the room and the resulting variability in the particle tracks following each cough, there is a natural variability in the results between simulations, even with the same mesh. Effort was made to reduce this effect by tracking more droplets per cough and more coughs per simulation, but it was not practical to do enough to completely remove this effect.

The results in the 2 m - 3 m volume are most sensitive to a change in the mesh as fewer droplets and particularly fewer large droplets reach this region. Therefore, the results are more sensitive to the unsteadiness in the room and the stochastic nature of the particle tracking.

As the variation in exposures between the baseline and refined meshes are reasonably small, it was decided to proceed with the standard mesh. However, it is recognised that there may be more confidence in the relative changes in the exposure further from the person than the absolute values.

- 1. **Time step size sensitivity**

CFD models at 20°C and 50% RH were run with three different sets of time step sizes to check for sensitivity to this parameter. All time step sizes were either multiplied or divided by a factor of two to produce the coarse and refined resolution simulations. The results are shown in Figure S3.

**
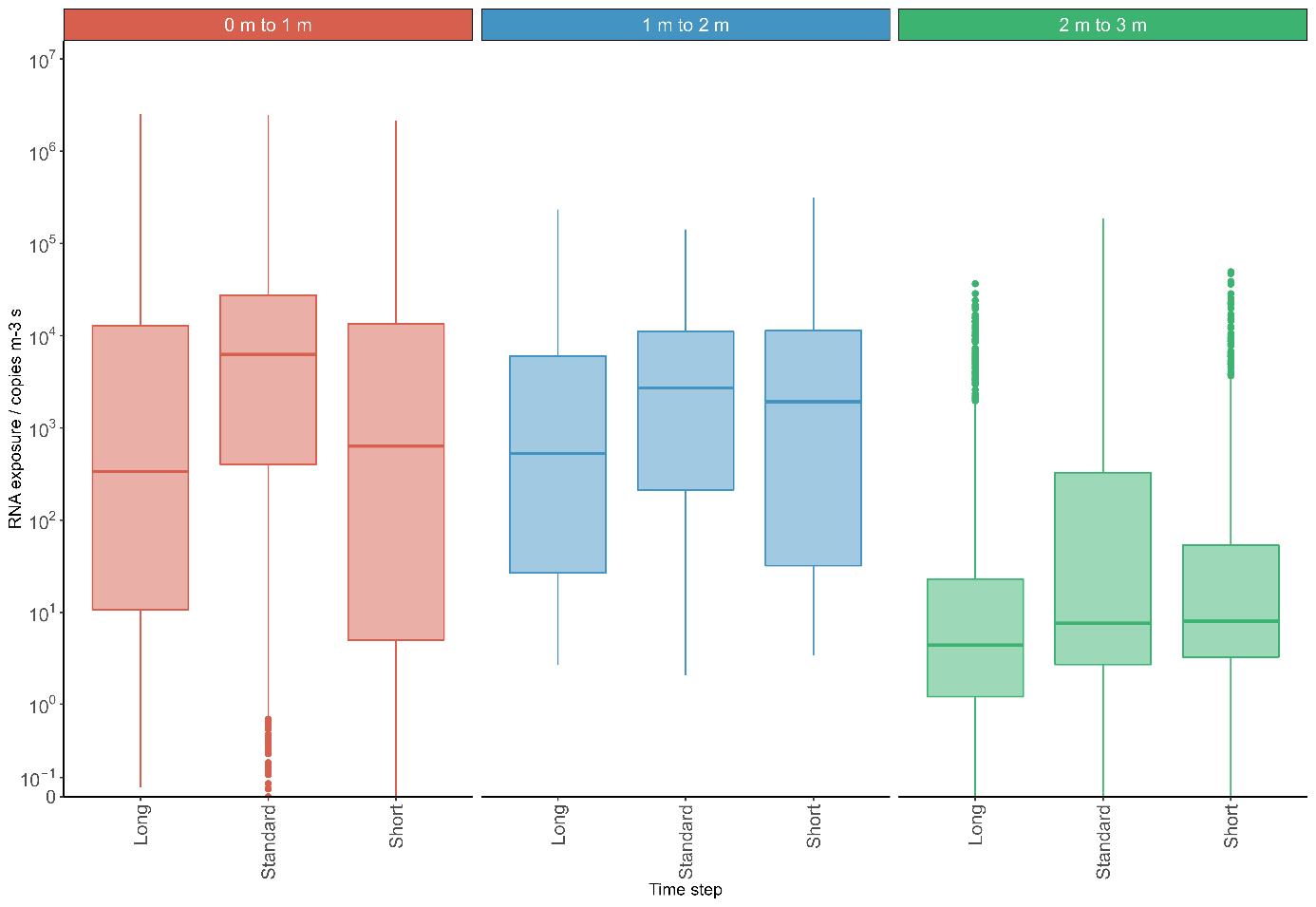
**

Figure S3 Time step size dependency box and whisker plots for exposure showing results for the long, standard and short time steps. Data is shown for three analysis volumes at increasing distance from the person and for 20°C and 50% RH only.

As with the mesh dependency, there was some dependence of the exposure distributions to the time step size. However, the change in the median exposure was relatively small when changing from the standard to the short time step sizes, particularly further from the person. The increased sensitivity close to the person was because the flow was fastest and the mesh was smallest in this region.

- 1. **Sensitivity to the number of coughs**

A CFD models at 20°C and 50% RH was run with ten coughs to see whether an increasing number of coughs (and subsequently more droplets) affected the exposure predictions. The resulting exposure distributions are shown in Figure S4.

**
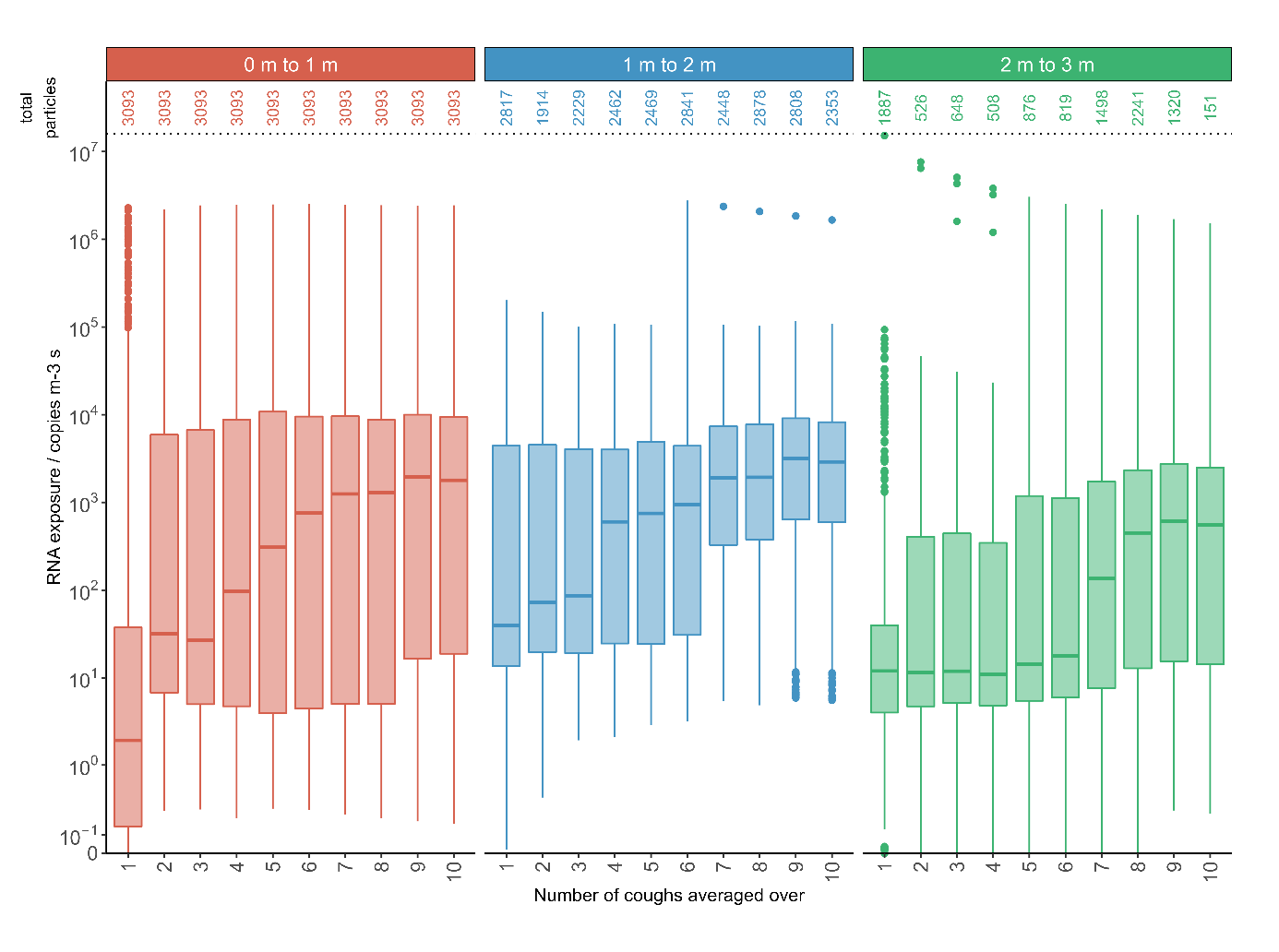
**

Figure S4 Box and whisker plots for exposure showing results for an increase number of cough. Data is shown for three analysis volumes at increasing distance from the person and for 20°C and 50% RH only. The numbers at the top of the graph show the number of particles that contribute to the exposures.

The results show that the exposure distributions have not converged fully after five coughs. The converged solution has been reached after six or seven coughs in the first volume, seven in the second and eight in the third. As the exposure distributions are not fully converged, the results of this work should be considered a snapshot of what could happen for any five coughs, rather than a perfect statistically stationary solution.

It should be noted that the exact convergence rate would depend on the order in which the coughs were averaged.

1. **Droplet size bins**

For the BLO model, the following size bins were used. This is for the BLO distribution that was scaled by a factor of ten.

| **Diameter (*d_0_*) / µm** | **Number of droplet per bin** |
| --- | --- |
| 0.25 | 2 |
| 0.36 | 13 |
| 0.52 | 45 |
| 0.76 | 184 |
| 1.10 | 580 |
| 1.58 | 1234 |
| 2.29 | 594 |
| 3.31 | 195 |
| 4.79 | 42 |
| 6.92 | 13 |
| 10.00 | 0 |
| 14.45 | 0 |
| 20.89 | 2 |
| 30.20 | 7 |
| 43.65 | 16 |
| 63.10 | 16 |
| 91.20 | 32 |
| 131.83 | 42 |
| 190.55 | 44 |
| 275.42 | 22 |
| 398.11 | 10 |

Table S1 Size bins for the BLO model.

1. **Representing received exposure in a CFD model**

The main weakness of the exposure-based approach used in this and other studies^4, 5^ is that the exposure reported is a function of the post-processing sub-volume size when the droplets or particles are not uniformly distributed within the sub-volumes. This effect will be most important close to the mouth of the coughing person where a stream of large droplets could pass through a small part of some of the sub-volumes. This is the challenge with using a continuous type exposure model to quantify a particle tracking model that uses discrete particles. The more particles that are released into the model, or the more the particles are mixed across the space, the smaller this error will be.

For an explicit representation of the susceptible person to be used to predict what that person is exposed to, requires a breathing person to be modelled^6^. To accurately calculate what they are inhaling, the full inhalation-exhalation cycle should be modelled. This can make the model more computationally expensive due to the extra mesh and temporal resolution required. An additional disadvantage of this approach is that it would not be practical to predict the exposure at lots of locations which are independent of each other, i.e., the flow around one susceptible person may affect the flow around a second person. Other studies have estimated the hazard or risk based on how much aerosol deposits on an explicitly modelled susceptible person^7, 8^.

1. **Tracer gas dispersion validation**

Predictions for the dispersion of a tracer gas in the meeting room being studied here were compared to data from an experiment described in Foat et al.^9^. In the experiment the total air change was 15.4 h^-1^, there was a recirculation fraction of 0.56, giving a fresh air change rate of 6.8 h^-1^. In the scenario used in the current work, the total air change was 5 h^-1^ and there was no recirculation. A tracer gas, propylene, was released in the corner of the room through a spherical diffuser (a device designed to reduce the momentum of a jet of gas). A cylinder of 2% propylene in nitrogen was used to deliver 0.04 m^3^ of the tracer at an approximately constant rate over a period of 180 s. The gas concentration was monitored in the room using photo-ionisation detectors (PIDs). Six of the PIDs were positioned 0.65 m above the ground with one at 1.95 m height. Some additional details of the experiment (and comparison using a different CFD package) are given in Foat et al. ^9^. The minimum, mean and maximum concentrations for all the locations at each time are shown in Figure S5. It should be noted that the comparison shown in the graph does not directly compare the concentration at one location in the CFD model with the same location in the experiment but represents some statistical characteristics of the concentration field.

The model was run with the same settings as described at the start of Section **Error! Reference source not found.**, but with steady-state airflow and isothermal conditions, as there were no heat sources in the room during the experiment. The turbulent Schmidt number for the tracer gas was set to 0.7.

As, shown in Figure S5, the CFD did not capture some of the unsteadiness in the concentration field, as would be expected when using a steady RANS modelling approach, but captured the trends well. The model over-predicted the highest concentrations (close to the gas release point). The model also predicted, perhaps, too much mixing, as can be seen from 400 s. However the relative separation of the experimental curves after this point, compared the separation of the model data, may be due to instrument calibration, rather than be a true effect of the gas dispersion.


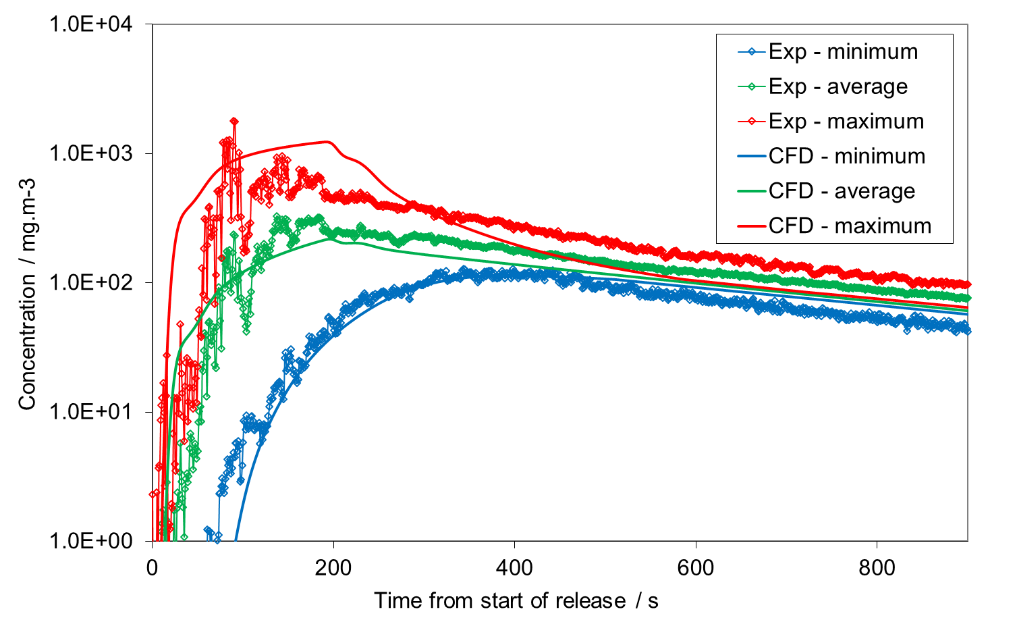


Figure S5 Maximum, average and minimum tracer concentrations across seven measurement locations from two experiments compared to predictions from the CFD model.

The model performance was assessed by calculating the geometric mean bias and geometric variance (as defined by Hanna et al.^10^) for all PID locations and time points when the measured concentration was greater than 1 mg·m^-3^. This was approximately the lowest concentration at which the PIDs were calibrated. The mean bias was 1.12 (compared to an ideal value of 1), which indicates that the model tended to slightly under-predict the experimental data. The geometric variance was 1.10 (compared to an ideal value of 1), which indicates that there was a small amount of scatter of the model data around the experimental data.

1. **Statistical analysis**

The following tables provide the output from the statistical analysis for the exposure data. Each table provides the results from a quantile regression model on the median log RNA exposure, whereby log RNA exposure was fitted as the dependent variable, and a combination of volume, temperature and/or RH fitted as the independent variables.

‘Ref. Cat.’ denotes the reference category selected for each independent variable. The other categories of each independent variable are then compared against this reference category in the regression, with the following results provided for each category:

- ‘Estimate’ – representing the difference in median log RNA exposure found for this category compared to the reference category (a positive value represents an increase in log RNA exposure, and a negative value a decrease in log RNA exposure).
- ‘Std. error’ – representing the associated standard error of the ‘Estimate’ accordingly.
- ‘z value’ – representing the difference of the estimate from 0 (the ‘Estimate’ divided by the ‘Std. error’); the larger the absolute z value, the more confident the category can be shown to have a statistically larger/smaller log RNA exposure compared to the reference category.
- ‘Pr(|z|)’ – representing the associated probability that the estimate is statistically larger/smaller compared to the reference category (and that the category has statistically larger/smaller log RNA exposure compared to the reference category accordingly).
  1. **Quantile regression models**
     1. **Initial assessment overall**

An initial model was formulated without any interactions to provide a basic understanding of the overall impact of volume, temperature and RH:

|  | **Estimate** | **Std. error** | **z value** | **Pr(>\|z\|)** |  |
| --- | --- | --- | --- | --- | --- |
| ***Count model coefficients*** | | | | | |
| **(Intercept)** | 8.04435 | 0.08552 | 94.0655 | < 2e-16 |  |
| **Volume** | | | | | |
| 0 m to 1 m (Ref. Cat.) | - | - | - | - |  |
| 1 m to 2 m | 0.01235 | 0.07703 | 0.16035 | 0.87261 |  |
| 2 m to 3 m | -5.2877 | 0.07903 | -66.904 | < 2e-16 |  |
| **Temperature** | | | | | |
| 16°C (Ref. Cat.) | - | - | - | - |  |
| 20°C | -0.0037 | 0.06972 | -0.0528 | 0.95787 |  |
| 28°C | 0.52896 | 0.06546 | 8.08028 | < 2e-16 |  |
| **Relative Humidity** | | | | | |
| 30% (Ref. Cat.) | - | - | - | - |  |
| 50% | -0.3371 | 0.07786 | -4.33 | 0.00002 |  |
| 70% | -0.401 | 0.06681 | -6.0019 | < 2e-16 |  |

Table S2 Quantile regression model summary comparing temperature, RH and volume, without interactions (complete data).

- - 1. **Full assessment overall**

Following a model selection process, in which more complex interactions are added to the model and compared with the simpler model form using likelihood ratio tests, a final model was formulated. This model included statistically significant interactions, which indicated that the degree in which the volume affected the median log RNA exposure was dependent, not only on the temperature but also on the RH. Likewise, this demonstrates that the degree in which the temperature affected the median log RNA exposure was dependent, not only on the volume but also on the RH, etc.

|  | **Estimate** | **Std. error** | **z value** | **Pr(>\|z\|)** |  |
| --- | --- | --- | --- | --- | --- |
| ***Count model coefficients*** | | | | | |
| **(Intercept)** | 3.36035 | 0.88207 | 3.80963 | 0.00014 |  |
| **Volume** | | | | | |
| 0 m to 1 m (Ref. Cat.) | - | - | - | - |  |
| 1 m to 2 m | 4.82107 | 0.88804 | 5.42887 | < 2e-16 |  |
| 2 m to 3 m | 2.30329 | 0.95846 | 2.40312 | 0.01627 |  |
| **Temperature** | | | | | |
| 16°C (Ref. Cat.) | - | - | - | - |  |
| 20°C | 4.21144 | 0.93369 | 4.51055 | 0.00001 |  |
| 28°C | 6.40278 | 0.88964 | 7.19701 | < 2e-16 |  |
| **Relative Humidity** | | | | | |
| 30% (Ref. Cat.) | - | - | - | - |  |
| 50% | -0.0068 | 1.25236 | -0.0054 | 0.99569 |  |
| 70% | 4.28057 | 0.89394 | 4.78843 | < 2e-16 |  |
| **Volume : Temperature Interactions** | | | | | |
| 1 m to 2 m : 20°C | -4.11 | 0.9454 | -4.3474 | 0.00001 |  |
| 1 m to 2 m : 28°C | -7.3529 | 1.01514 | -7.2432 | < 2e-16 |  |
| 2 m to 3 m : 20°C | -5.9518 | 0.90299 | -6.5912 | < 2e-16 |  |
| 2 m to 3 m : 28°C | -9.8825 | 0.96751 | -10.214 | < 2e-16 |  |
| **Volume : RH Interactions** | | | | | |
| 1 m to 2 m : 50% | -0.3675 | 1.26142 | -0.2913 | 0.77082 |  |
| 1 m to 2 m : 70% | -2.448 | 1.33171 | -1.8382 | 0.06605 |  |
| 2 m to 3 m : 50% | -4.8299 | 0.91001 | -5.3076 | < 2e-16 |  |
| 2 m to 3 m : 70% | -7.6166 | 0.97084 | -7.8454 | < 2e-16 |  |
| **Temperature : RH Interactions** | | | | | |
| 20°C : 50% | 1.51149 | 1.29404 | 1.16805 | 0.24281 |  |
| 28°C : 50% | -1.3402 | 1.27571 | -1.0505 | 0.29349 |  |
| 20°C : 70% | -4.1268 | 0.97069 | -4.2514 | 0.00002 |  |
| 28°C : 70% | -5.8425 | 0.90896 | -6.4276 | < 2e-16 |  |
| **Volume : Temperature : RH Interactions** | | | | | |
| 1 m to 2 m : 20°C : 50% | -1.98163 | 1.31317 | -1.50904 | 0.13131 |  |
| 2 m to 3 m : 20°C : 50% | -0.2498 | 1.38028 | -0.18098 | 0.85639 |  |
| 1 m to 2 m : 28°C : 50% | 1.10078 | 1.30302 | 0.84479 | 0.39824 |  |
| 2 m to 3 m : 28°C : 50% | 6.38069 | 1.53601 | 4.15407 | 0.00003 |  |
| 1 m to 2 m : 20°C : 70% | 4.0642 | 1.00445 | 4.04621 | 0.00005 |  |
| 2 m to 3 m : 20°C : 70% | 7.61708 | 1.05886 | 7.19366 | < 2e-16 |  |
| 1 m to 2 m : 28°C : 70% | 5.85195 | 0.93774 | 6.24048 | < 2e-16 |  |
| 2 m to 3 m : 28°C : 70% | 9.536 | 0.99166 | 9.61621 | < 2e-16 |  |

Table S3 Quantile regression model summary comparing temperature, RH and volume, with interactions (complete data).

As significant interactions between volume, temperature and RH were found, three additional models were formulated accordingly for each volume separately to better understand and illustrate the effect of temperature and RH within each volume.

- - 1. **Assessing by temperature for volume 0 m to 1 m**

|  | **Estimate** | **Std. error** | **z value** | **Pr(>\|z\|)** |
| --- | --- | --- | --- | --- |
| **Count model coefficients** | | | | |
| **(Intercept)** | 6.42185 | 0.42647 | 15.05809 | < 2e-16 |
| **Temperature** | | | | |
| 16°C (Ref. Cat.) | - | - | - | - |
| 20°C | 1.81141 | 0.43882 | 4.12787 | 0.00004 |
| 28°C | 2.25768 | 0.43334 | 5.20995 | < 2e-16 |

Table S4 Quantile regression model summary comparing temperature (0 m - 1 m volume only).

- - 1. **Assessing by temperature and RH for volume 1 m to 2 m**

|  | **Estimate** | **Std. error** | **z value** | **Pr(>\|z\|)** |
| --- | --- | --- | --- | --- |
| ***Count model coefficients*** | | | | |
| **(Intercept)** | 8.2426 | 0.0893 | 92.30019 | < 2e-16 |
| **Temperature** | | | | |
| 16°C (Ref. Cat.) | - | - | - | - |
| 20°C | -0.0674 | 0.10877 | -0.61961 | 0.53554 |
| 28°C | 0.39682 | 0.10211 | 3.88626 | 0.0001 |
| **Relative Humidity** | | | | |
| 30% (Ref. Cat.) | - | - | - | - |
| 50% | -0.58773 | 0.10627 | -5.53052 | < 2e-16 |
| 70% | -0.55867 | 0.10423 | -5.36011 | < 2e-16 |

Table S5 Quantile regression summary comparing temperature and RH (1 m to 2 m volume only).

- - 1. **Assessing by temperature and RH for volume 2 m to 3 m**

|  | **Estimate** | **Std. error** | **z value** | **Pr(>\|z\|)** |  |
| --- | --- | --- | --- | --- | --- |
| ***Count model coefficients*** | | | | | |
| (Intercept) | 3.12649 | 0.15019 | 20.81761 | <2e-16 |  |
| **Temperature** | | | | | |
| 16°C (Ref. Cat.) | - | - | - | - |  |
| 20°C | -0.68261 | 0.1343 | -5.08265 | <2e-16 |  |
| 28°C | -0.27902 | 0.13805 | -2.0212 | 0.04332 |  |
| **Relative Humidity** | | | | | |
| 30% (Ref. Cat.) | - | - | - | - |  |
| 50% | -0.24823 | 0.15286 | -1.62388 | 0.10447 |  |
| 70% | -0.32629 | 0.13546 | -2.40871 | 0.01605 |  |

Table S6 Quantile regression model summary comparing temperature and RH (2 m to 3 m volume only).

- 1. **Summary Statistics**

| **Volume** | **Temperature** | **RH** | **Median (IQR) / copies·s·m-3** |
| --- | --- | --- | --- |
| 0 m to 1 m | 16°C | 30% | 28.4 (3.1, 7861.1) |
|  |  | 50% | 23.8 (1.3, 5690) |
|  |  | 70% | 2093.4 (12.0, 9710.3) |
|  | 20°C | 30% | 1938.6 (6.6, 22442.4) |
|  |  | 50% | 8730.6 (994.6, 23230.4) |
|  |  | 70% | 2262.8 (12.9, 10645.2) |
|  | 28°C | 30% | 17364.5 (2666, 52010.6) |
|  |  | 50% | 4573.6 (34.7, 22479.3) |
|  |  | 70% | 3606 (43.9, 13007.5) |
| 1 m to 2 m | 16°C | 30% | 3553 (526.0, 13724.1) |
|  |  | 50% | 2470.8 (557.8, 7179.8) |
|  |  | 70% | 2065.3 (172.2, 7928.9) |
|  | 20°C | 30% | 3925.7 (642.3, 13913.9) |
|  |  | 50% | 1687.1 (26.4, 7889.8) |
|  |  | 70% | 2142.6 (166.9, 9470) |
|  | 28°C | 30% | 5601.3 (1065.8, 21834.5) |
|  |  | 50% | 3069.7 (275.8, 16853.9) |
|  |  | 70% | 3256.7 (307.5, 9013.2) |
| 2 m to 3 m | 16°C | 30% | 269.9 (10.9, 2997.1) |
|  |  | 50% | 24.6 (7.6, 1148.7) |
|  |  | 70% | 10.2 (5.2, 336.3) |
|  | 20°C | 30% | 12.2 (2.8, 904.2) |
|  |  | 50% | 3.6 (1.4, 124.4) |
|  |  | 70% | 14.5 (4.0, 1522.8) |
|  | 28°C | 30% | 8.7 (3.6, 586.7) |
|  |  | 50% | 100.4 (6.3, 2756.6) |
|  |  | 70% | 12.7 (5.0, 985.7) |

Table S7 RNA Exposure summary statistics by volume, temperature and RH (complete data).

| **Volume** | **Median (IQR) / copies·s·m^-3^** |
| --- | --- |
| 0 m to 1 m | 2940.0 (12.9, 17980.0) |
| 1 m to 2 m | 2938.0 (347.0, 11161.0) |
| 2 m to 3 m | 13.8 (4.3, 1151.0) |

Table S8 RNA Exposure summary statistics by volume (complete data).

| **Volume** | **RH** | **Median (IQR) / copies·s·m^-3^** |
| --- | --- | --- |
| 0 m to 1 m | 30% | 3095.0 (8.5, 29071.0) |
|  | 50% | 3154.0 (13.9, 17872.0) |
|  | 70% | 2647.0 (18.9, 10914.0) |
| 1 m to 2 m | 30% | 4179.0 (696.0, 15868.0) |
|  | 50% | 2283.0 (223.0, 9361.0) |
|  | 70% | 2488.0 (218.0, 8868.0) |
| 2 m to 3 m | 30% | 16.2 (4.7, 1474.0) |
|  | 50% | 13.9 (3.4, 1161.0) |
|  | 70% | 12.0 (4.7, 838.0) |

Table S9 RNA Exposure summary statistics by volume and RH (complete data).

| **Volume** | **Temperature** | **Median (IQR) / copies·s·m^-3^** |
| --- | --- | --- |
| 0 m to 1 m | 16°C | 504.0 (3.6, 7521.0) |
|  | 20°C | 3722.0 (18.0, 19236.0) |
|  | 28°C | 5890.0 (278.0, 28071.0) |
| 1 m to 2 m | 16°C | 2602.0 (424.0, 9507.0) |
|  | 20°C | 2408.0 (208.0, 10025.0) |
|  | 28°C | 3789.0 (444.0, 13897.0) |
| 2 m to 3 m | 16°C | 19.8 (7.1, 1325.0) |
|  | 20°C | 9.2 (2.3, 744.0) |
|  | 28°C | 13.5 (4.7, 1378.0) |

Table S10 RNA Exposure summary statistics by volume and temperature (complete data).

1. **References**
2. Singanayagam A, et al. Community transmission and viral load kinetics of the SARS-CoV-2 delta (B. 1.617. 2) variant in vaccinated and unvaccinated individuals in the UK: a prospective, longitudinal, cohort study. The Lancet Infectious Diseases. 2021.
3. Chen PZ, Bobrovitz N, Premji Z, Koopmans M, Fisman DN, Gu FX. Heterogeneity in transmissibility and shedding SARS-CoV-2 via droplets and aerosols. Elife. 2021;10:e65774.
4. Roque M, Proudfoot K, Mathys V, Yu S, Krieger N, Gernon T, Gokli K, Hamilton S, Cook C, Fong Y. A review of nasopharyngeal swab and saliva tests for SARS‐CoV‐2 infection: Disease timelines, relative sensitivities, and test optimization. Journal of surgical oncology. 2021;124(4):465-75.
5. Liu H, He S, Shen L, Hong J. Simulation-based study on the COVID-19 airborne transmission in a restaurant. medRxiv. 2020.
6. Wan MP, Sze To GN, Chao CY, Fang L, Melikov A. Modeling the fate of expiratory aerosols and the associated infection risk in an aircraft cabin environment. Aerosol Science and Technology. 2009;43(4):322-43.
7. Deng Z, Chen Q. What is suitable social distancing for people wearing face masks during the COVID‐19 pandemic? Indoor air. 2021;00:1-15.
8. Li H, Leong FY, Xu G, Ge Z, Kang CW, Lim KH. Dispersion of evaporating cough droplets in tropical outdoor environment. Physics of Fluids. 2020;32(11):113301.
9. Yang X, Ou C, Yang H, Liu L, Song T, Kang M, Lin H, Hang J. Transmission of pathogen-laden expiratory droplets in a coach bus. Journal of hazardous materials. 2020 Oct 5;397:122609.
10. Foat T, Drodge J, Nally J, Parker S. A relationship for the diffusion coefficient in eddy diffusion based indoor dispersion modelling. Building and Environment. 2020;169:106591.
11. Hanna SR, Hansen OR, Dharmavaram S. FLACS CFD air quality model performance evaluation with Kit Fox, MUST, Prairie Grass, and EMU observations. Atmospheric Environment. 2004;38(28):4675-87.

© Crown copyright (2022), Dstl, HSE. This material is licensed under the terms of the Open Government Licence except where otherwise stated. To view this licence, visit http://www.nationalarchives.gov.uk/doc/open-government-licence/version/3 or write to the Information Policy Team, The National Archives, Kew, London TW9 4DU, or email: [psi@nationalarchives.gov.uk](mailto:psi@nationalarchives.gov.uk)
